# Geochemistry and mineralogy of the Oligo-Miocene sediments of the Valley of Lakes, Mongolia

**DOI:** 10.1007/s12549-016-0268-6

**Published:** 2017-02-13

**Authors:** Sylvain Richoz, Andre Baldermann, Andreas Frauwallner, Mathias Harzhauser, Gudrun Daxner-Höck, Dietmar Klammer, Werner E. Piller

**Affiliations:** 1 Institute of Earth Sciences, Nawi Graz, Graz University, Heinrichstraße 26, 8010 Graz, Austria; 2 Institute of Applied Geosciences, Graz University of Technology, Rechbauerstr. 12, 8010 Graz, Austria; 3 Natural History Museum Vienna, Burgring 7, 1010 Vienna, Austria

**Keywords:** Mongolia, Oligocene, Miocene, Climate change, Illitization, Paleosol

## Abstract

The Valley of Lakes is approximately a 500-km elongate depression in Central Mongolia, where Eocene to Miocene continental sediments are long known for their outstanding fossil richness. The palaeontological record of this region is an exceptional witness for the evolution of mammalian communities during the Cenozoic global cooling and regional aridification. In order to precisely elucidate the climatic evolution of the region, we studied the mostly siliciclastic sediments with several levels of paleosols for their sedimentology, mineralogy, major and trace element composition and δ^13^C and δ^18^O composition. The obtained results show that temperate hydrothermal fluids induced a strong illitization of the fluvial and lacustrine sediments. This finding contradicts the current conceptual view that the fine fraction of the sediments is of aeolian origin. Moreover, the diagenetic growth of illite resulted in a strong overprinting of the sediments and, subsequently, largely disturbed the pristine mineralogical and geochemical composition of the sediments that could have carried any palaeo-climatic information. An exception is the δ^13^C (and δ^18^O) isotope values of authigenic carbonate found in calcrete horizons that still record the ambient climatic conditions prevailing during paleosol formation. Our novel δ^13^C and δ^18^O record suggests an early Oligocene aridification in Central Asia at ∼31 Ma, whereas the Oligocene glacial maximum shows no increase in aridification. A second, regional-scale aridification occurs at ~25 Ma and corresponds to a late Oligocene marked mammalian turnover in the Valley of Lakes sediments.

## Introduction

The Cenozoic climate cooling is well recorded in marine sediments; however, its repercussion on the evolution of continental ecosystems on a global and regional scale remains questionable, as appropriate continental sediment records are rather scarce. Central Asia is certainly a key area for investigating the impact of this global cooling event and particularly the Valley of Lakes in Mongolia. This ~500-km-long sedimentary basin comprises an almost continuous succession of Eocene to Miocene continental sediments that are charac-terised by an outstanding Oligo-Miocene fossil record (see Harzhauser et al. 2016; Daxner-Höck et al. 2017 and other contributions of this special issue and references therein for a palaeontological overview). Three prominent flood basalts crop out at distinct levels in the sedimentary succession and represent stratigraphic marker beds (Höck et al. 1999). Absolute dating of these basalt levels (Höck et al. 1999), fine biostratigraphy of small mammals (Daxner-Höck et al. 2010; Daxner-Höck et al. 2017, this issue) and magnetostratigraphy (Sun and Windley 2015) allow good correlation between the different studied sections and a well-established stratigraphy of the Valley of Lakes sediments (see Harzhauser et al. 2017, this issue and Daxner-Höck et al. 2017, this issue for a more detailed discussion on the stratigraphy). In this contribution, we revise the current model proposed for the climatic and mineralogical evolution of the Valley of Lakes sediments based on mineralogical, major and trace elemental and δ^13^C and δ^18^O isotope signatures of the sediments.

The Eocene-Oligocene Transition (EOT, ~34 Ma) is the first major cooling phase during the long-term transition from Cenozoic greenhouse to icehouse climate (Zachos et al. 2001; Lear et al. 2008). It is well recorded in δ^18^O profiles established on the basis of deep-sea benthic foraminifers (Zachos et al. 2001; Coxall et al. 2005). In Central Asia, this transitional phase started in the late Eocene and is marked by a sudden acceleration of the aridification (Dupont-Nivet et al. 2007; Xiao et al. 2010; Abels et al. 2011; Bosboom et al. 2014; Li et al. 2016) and by a dramatic faunal turnover, the “Mongolian Remodeling” (Meng and McKenna 1998; Kraatz and Geisler 2010; Harzhauser et al. 2016). The EOT aridification has been assigned to coupled global cooling, stepwise retreat of the proto-Paratethys Sea (Abels et al. 2011; Bosboom et al. 2014; Caves et al. 2015) and intensified uplift of the Tibetan and Mongolian Plateaus (Wang et al. 2012; Caves et al. 2014). The global climate in the Oligocene is characterised by glacial–interglacial cycles responding to astronomical forcing (Wade and Pälike 2004; Retallack et al. 2004; Pälike et al. 2006; Xiao et al. 2010). During this period, the more pronounced cooling phase, which is the Oligocene Glacial Maximum (OGM), occurred around 28–27 Ma and could correspond to a significant uplift phase of the Himalayan–Tibetan region accompanied by an increase in the rate of silicate weathering (Li et al. 2005), coevolution of grasses and grazers (Retallack 2013) and/or by an important volcanic activity at La Garita caldera (Colorado) (Phillips and Matchan 2013). This OGM is followed by the global Late Oligocene Warming, which is terminated by a renewed glacial episode at the Oligocene–Miocene transition (Miller et al. 1991; Paul et al. 2000). Based on sedimentological evidence, this latter cooling event is thought to have corresponded to a second prominent pulse of aridification expressed by the widespread formation of deserts in Central Asia (Guo et al. 2002, 2008; Sun et al. 2010).

At present, there is compelling evidence for two major aridification pulses at the Eocene–Oligocene and Oligocene–Miocene transition in Central Asia that could be related to global cooling events. To date, however, the correlation of the numerous climatic variations observed in the Oligocene marine record with the sedimentological and palaeontological data of Mongolia (presented in this issue and by Harzhauser et al. 2016) remains poorly constrained. We present here a novel and comprehensive mineralogical and (isotope) geochemical dataset of the highly fossiliferous Oligo-Miocene sediments from the Valley of Lakes (Mongolia) and critically re-evaluate the palaeo-climatic evolution of this famous study site.

## Geological setting and lithostratigraphy

**Fig. 1:**
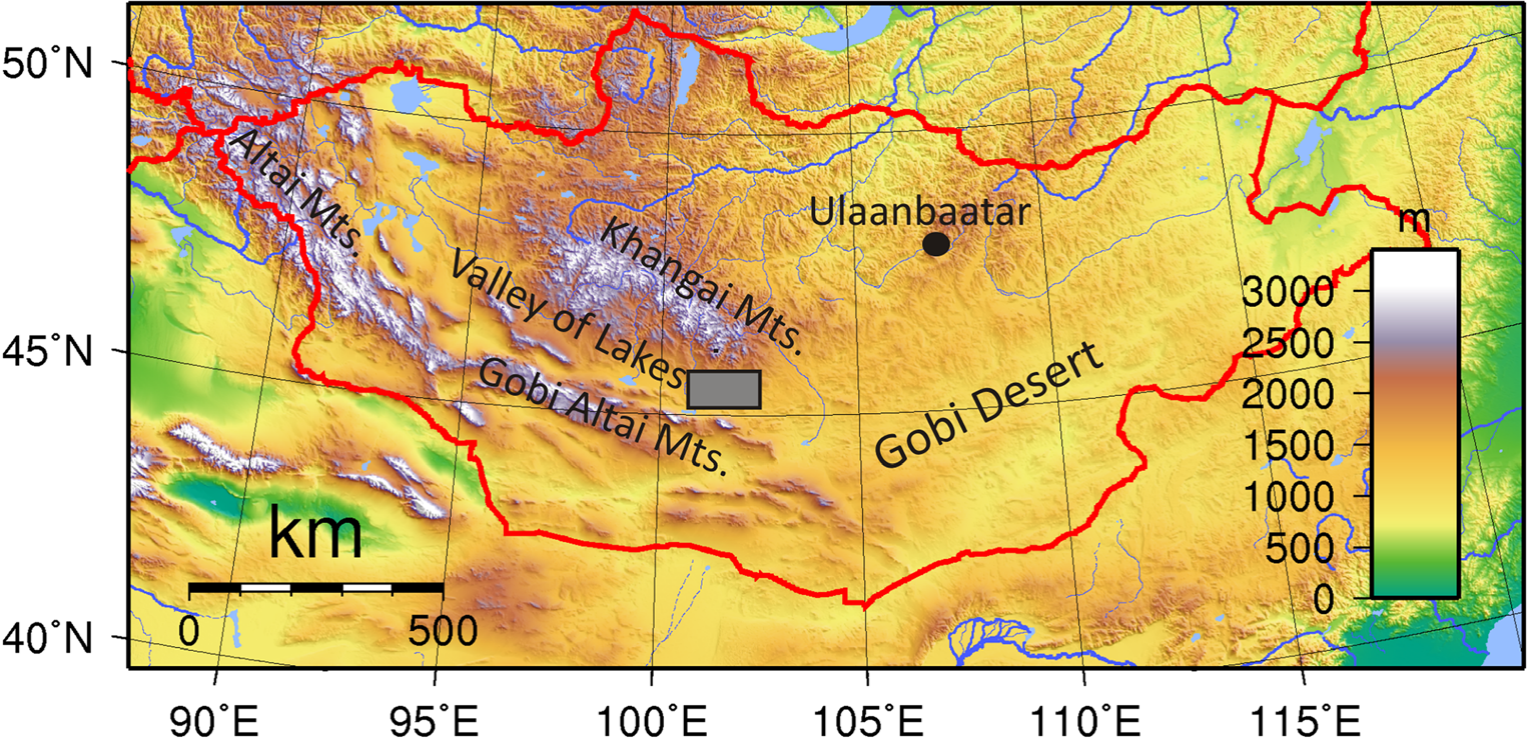
Location of the Taatsiin Gol region (*rectangle*), which is part of the Valley of Lakes in Central Mongolia. *Colour ban* indicates altitude in metres

**Fig. 2:**
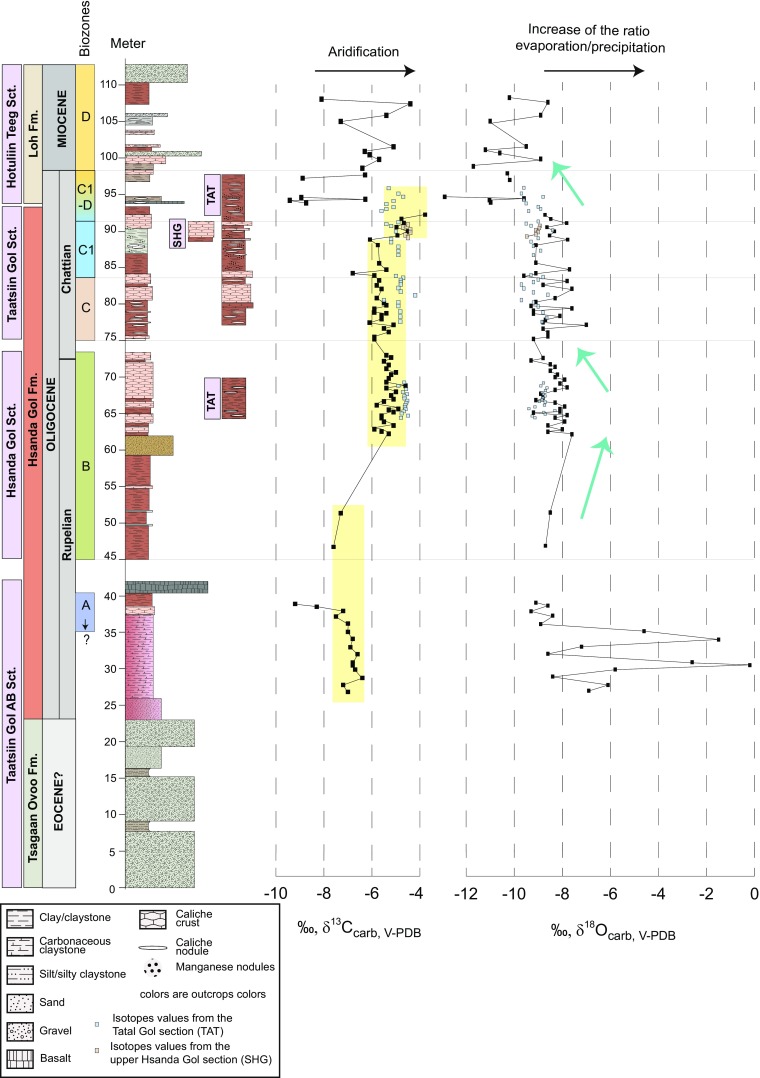
Stratigraphic overview of the investigated sedimentary succession from the Taatsiin Gol region including the δ^18^O and δ^13^C isotope profiles of soil carbonate preserved in calcrete horizons. The stratigraphic and biozone classification follows Daxner-Höck et al. 2017 in this issue. SHG is the upper part of the Hsanda Gol section. TAT corresponds to the Tatal Gol section

The Valley of Lakes is an elongate, ~500 km long, ESE-WNW striking Cis-Altai depression, located in Central Mongolia (Fig. 1). This sedimentary basin is situated between the Gobi Altai Mountains in the south and the Khangai Mountains in the north. It is filled with continental sedimentary rocks of Cretaceous to Quaternary age, deposited above the Proterozoic–Paleozoic basement (Höck et al. 1999; Daxner-Höck et al. 2010, 2014). The mostly siliciclastic Eocene to Miocene sediments are exposed along steep cliffs of mostly dry river beds of the Taatsiin Gol Basin and are long known for their extraordinary fossil richness (e.g. Daxner-Höck et al. 1997, 2010, 2014; Höck et al. 1999; Daxner-Höck and Badamgarav 2007; and other contributions of this special issue). The nomenclature, type area, reference profiles and lithological and sedimentological description of the Eocene Tsagaan Ovoo Fm., the Oligocene Hsanda Gol Fm. and the Miocene Loh Fm. are extensively described by Höck et al. (1999) and Daxner-Höck et al. (2017) in this issue. Three groups of basalt horizons are exposed in the sedimentary succession (Fig. 2) and provide the basis for the chronostratigraphic classification of the Oligo-Miocene sediments (Höck et al. 1999). From the bottom to the top of the sequence, the lower Oligocene basalt I group (~30 to 32 Ma), the upper Oligocene basalt II group (~25–29 Ma) and the middle Miocene basalt III (~13 Ma) (Höck et al. 1999; Daxner-Höck et al. 2014).

For this study five sections, all located in the Taatsiin Gol und Taatssin Tsagaan Nuur regions of the Valley of Lakes, have been chosen, namely the Taatsiin Gol right (TGR-AB) and south (TGR-C), the Hsanda Gol (SHG-D), the Tatal Gol (TAT-E) and the Hotuliin Teeg (HTE). We concentrate our study on the upper Eocene to lower Miocene interval in these sections (~35 to ~21 Ma including basalt I and basalt II), leaving the main Miocene part. This covers the upper part of the Eocene Tsagaan Ovoo Fm., the Oligocene Hsanda Gol Fm. and the lowermost part of the Miocene Loh Fm. A comprehensive stratigraphic profile is shown in Fig. 2 and represents a rather continuous sedimentation even if the gap cannot be excluded. The geographic details, sedimentological characteristics and stratigraphic position of these sections are reported and discussed by Daxner-Höck et al. 2017 and Harzhauser et al. 2017 in this issue.

### Petrography and sedimentology

The coarse clastic Tsagaan Ovoo Fm. is about 150 m thick and comprises predominantly white-greyish, massive to cross-bedded sand and gravel bodies, in which greyish-yellow-green to reddish-brown clay and silt layers are intercalated (Fig. 2). The gravel beds, which have been interpreted as debris flow deposits, following the facies classification of Miall (1996) for fluvial sediments, are up to 5 m thick, poorly sorted and mostly without visible sedimentary structures. Finer clastic beds occur towards the top of this sequence and show trough and planar cross-bedding with up to 1 m thickness, besides lamination, ripples, inverse to normal grading, horizontal bedding and channel fills. These sedimentary structures are indicative of gravel- to sand-bed braided river deposits associated with N to S propagating alluvial fans in a tectonically active basin margin (Miall 1990, 1996; Höck et al. 1999). Bioturbation, roots and plant debris point to the local formation of paleosols. Magnetostratigraphic studies reveal a late Eocene age of the Tsagaan Ovoo Fm. (Kraatz and Geisler 2010).

The Oligocene Hsanda Gol Fm. is characterised by a high fossil content and comprises poorly sorted, brick-red to reddish-brown clay with grey sandstone in some places (Fig. 2). The massive, mostly horizontally bedded, clayey and silty beds show non-erosive boundaries and frequently contain irregular marly layers, besides cm- to dm-sized nodules of soil material cemented with secondary calcite. These features have been interpreted as calcrete (also called caliche) developed in paleosols under arid to semiarid climate (McPherson 1979; Reineck and Singh 1986). Root-traces, plant debris and burrows occur in these layers. More rarely, sand and granule lenses can be found, which have been attributed to lacrustrine or fluvial reworking processes. In section SHG, a horizon with greyish-coloured sands and gravels forms a notable interval. The depositional environment was interpreted as semiarid, open steppe with ephemeral rivers and playa lakes (Daxner-Höck et al. 2010; Sun and Windley 2015). At nearly 40–41 m and between 94–100 m, the stratigraphic important basalt I and II groups crop out (Fig. 2).

The widespread Loh Fm. is up to 150 m thick and is characterised by a complex lithological variability (Fig. 2), comprising alternating poorly sorted, greenish-yellow-red and widely structure-less sandy clays with pebbles and greyish-white to reddish-brown, trough to planar cross-bedded clayey sands and gravels of fluvial origin (Miall 1990, 1996). Channel and scour infillings, fining upward sequences with inverse to normal grading, small ripple marks and overbank fines can be found and have been interpreted as abandoned channel and waning flood deposits of either a shallow, gravel-bed braided river or a perennial flowing sand-bed braided river with ephemeral character (Reineck and Singh 1986; Miall 1996). Sediments of the Loh Fm. are partly interfingering with those from the Hsanda Gol Fm. and are of late Oligocene to early Miocene age. Burrows and plant debris occur within the fossiliferous calcrete horizons. Imbricated gravel beds, cross-bedded sands and flow structures in the basalt group III (not represented in Fig. 2) point to a palaeo-current direction from N to S (Höck et al. 1999).

### Paleosol horizons

Paleosol horizons are frequently developed within the Hsanda Gol and Loh fms. (Fig. 2). These paleosols can be easily identified in the field due to their close association with calcrete horizons (see the above petrological description). Calcretes are ubiquitous features of arid to semiarid and subhumid landscapes, where net evaporation typically exceeds net precipitation (Retallack 1994). The formation of calcretes is generally associated with soil-forming (pedogenic) processes or related to a prolonged interaction with meteoric solutions and/or groundwater (Khadikikar et al. 2000). In the Hsanda Gol and Loh fms., they occur in the form of either laminar (continuous) or irregular and patchy (discontinuous) carbonate nodules and crusts of whitish to pinkish colour. Both types of calcrete consist of almost pure crypto- to microcrystalline calcite. Nodules are often mottled, occasionally arranged in honeycomb-like structures and may contain traces of roots, as well as orange-brown (limonitic) Fe-(oxy)hydroxides and greyish-black Mn-oxides. In some sections, the extensive formation of calcrete affected several metres of the underlying sediments (e.g. TGR-C/10, TGR-C/19, TAT/14-16), suggesting a considerable time of paleosol formation. It is generally accepted that the ambient climatic conditions prevailing during the formation of paleosols may be best preserved in the calcrete horizons and, in particular, in the isotopic and major and trace element composition of the authigenic carbonate. We collected sediment samples from each layer which differed from the underlaying layer by colour, composition, structure, fossil content, etc. All calcrete horizons were sampled in order to identify potential (palaeo-) climatic changes throughout the Oligo-Miocene transition recorded in the Valley of Lakes sediments.

## Methods

### X-ray fluorescence and the chemical index of alteration

The major and minor element composition of rock samples was analysed with a Philips PW2404 wavelength dispersive X-ray fluorescence (XRF) spectrometer. About 0.8 g of finely ground sample was heated to 1050 °C to remove volatiles, such as CO_2_ and H_2_O, and then the loss on ignition (LOI) was determined by gravimetric analysis. The residual was fused at 1200 °C using 4 g of LiBO_2_ as the fluent agent. The tablets produced were run together with a range of USGS standards. The analytical error is ±0.5 wt% for the major elements.

Geochemical estimations of weathering intensity and paths were derived from XRF data, assuming that changes in the bulk rock composition reflect distinct alteration features, i.e. hydrolysis and K-metasomatism. For instance, the progressive transformation of feldspar to more stable clay minerals, such as illite and kaolinite, at ambient environmental conditions can be traced by following changes in the ratio of immobile Al_2_O_3_ to the more mobile cations K^+^, Na^+^ and Ca^2+^ expressed as oxides. The quantitative measure of chemical weathering is the Chemical Index of Alteration (CIA, Nesbitt and Young 1982), which is defined as: CIA = (Al_2_O_3_·(Al_2_O_3_ + Na_2_O + K_2_O + CaO*)^−1^)·100. Note that CaO present as carbonate was subtracted from the bulk CaO content (on the basis of TIC values) to obtain CaO* of the silicate fraction. CaO present as phosphate was not considered in the calculation of the CIA values because it increases the CIA by ~2 units if all P_2_O_5_ is assigned to apatite.

### Carbon and oxygen isotopes

The carbonate present in the calcrete horizons was analysed for their stable δ^13^C and δ^18^O isotope composition in order to evaluate potential palaeo-climatic trends recorded in the paleosols. All samples were crushed and analysed as bulk rock. For some delicate samples, carbonate nodules and crusts were separated by hand using a dental drill. Sample powders were reacted with 102% phosphoric acid at 70 °C in a Kiel II automated reaction system, and the evolved CO_2_ was analysed with a ThermoFinnigan Mass spectrometer MAT Delta at the University of Graz. The δ^13^C and δ^18^O values are corrected according to the NBS19 standard and reported in per mill (‰) relative to the Vienna-PeeDee Belemnite (V-PDB) standard. The analytical precision is <0.05‰ for δ^13^C and <0.1‰ for δ^18^O, respectively.

### X-ray diffraction

X-ray diffraction (XRD) patterns were recorded for quantitative mineral phase analyses using a PANalytical X’Pert PRO diffractometer (Co-Kα radiation) operated at 40 kV and 40 mA and equipped with a Scientific X’Celerator detector, 0.5° antiscattering and divergence slits, spinner stage, primary and secondary soller and automatic sample changer. Representative rock samples were first crushed in a McCrone micronizing mill for 8 min, together with 10% zincite as the internal standard. Subsequently, randomly oriented samples were prepared using the top loading technique. The specimens were examined over the range 4–85 2θ using a step size of 0.008°2θ/s and a count time of 40 s/step. Rietveld-based mineral quantifications were carried out with the PANanalytical X`Pert Highscore Plus software and its implemented pdf-2 database. Assuming the idealised compositions for quartz, albite, orthoclase, illite, hematite and calcite (Baldermann et al. 2013), the accuracy of these results was verified by comparison with mass–balance calculations based on bulk rock XRF data. All mineral phases with an abundance below 1 wt% (chlorite, kaolinite, vermiculite, halite, amphibole and zeolite, if present) were not considered in the quantitative mineral analyses. The deviation between XRD and XRF results was <3 wt%.

The clay mineral fraction (<2 μm) was further charac-terised using XRD analysis of oriented preparations performed on a Phillips PW 1830 diffractometer (Cu-Kα radiation, 40 kV and 30 mA) outfitted with automatic slits, a graphite monochromator and a scintillation counter. About 50 mg of the sample was mixed with 5 mL of deionised water, following dispersion for 10 min in an ultrasonic bath. The clay-in-suspension was then sucked through a ceramic tile of about 4 cm^2^ in order to produce oriented specimens (Baldermann et al. 2014). These clay films were X-rayed from 3 to 30° 2θ with a step size of 0.02° 2θ and a count time of 2 s per step, each at air-dried states and after the solvation of the clay matter with ethylene glycol (EG) and heating of the preparations to 550 °C for 1 h. The percentage of illite layers (%I) in illite–smectite (I-S) mixed layered clay minerals was determined based on EG-solvated patterns with an analytical precision of ±5% (Baldermann et al. 2013).

### Scanning electron microscopy

The mineralogy, particle form and particle shape of authigenic versus detrital (clay) minerals were studied by scanning electron microscopy (SEM) using a GEMINI®technology Zeiss Ultra 55 microscope at the FELMI-ZFE (TU Graz). Representative rock chips were therefore prepared on stubs, and the specimens were sputtered with Au/Pd to reduce charging. The SEM is equipped with a high efficiency in-lens secondary electron (SE) detector and an EDAX Si(Li)-detector for energy-dispersive X-ray spectrometry (EDS) analysis and was operated at an accelerating voltage of 5 kV.

## Results

In this chapter, special focus is placed on the identification and characterisation of unidirectional alteration and chemical weathering pathways of the sediments (and intercalated basalts). Such processes have to be considered for the interpretation of palaeo-climatic trends that are based on mineralogical and (isotope) chemical signatures of rock samples discussed below.

### Bulk and clay mineralogy

**Fig. 3:**
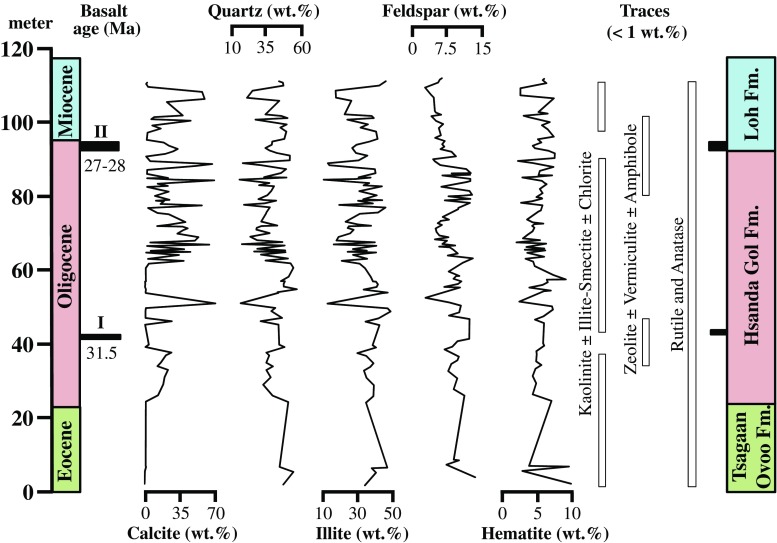
Bulk mineralogy of sediments from the Tsagaan Ovoo Fm., Hsanda Gol Fm. and Loh Fm. (Valley of Lakes, Central Mongolia) based on XRD data. The location and age of the prominent basalt horizons I to II are shown

The bulk mineralogy of the investigated sedimentary succession is displayed in Fig. 3. Quartz, sheet silicates, calcite, hematite, plagioclase and minor orthoclase are by far the major constituents. Considering the particle size, more than 50% of the population belongs to the clay fraction, whereas less than 1% belongs to the sand fraction or coarser (Höck et al. 1999). In contrast to the mineralogical composition previously reported by Höck et al. (1999), no indication of dolomite, ankerite, anhydrite and pyrite was found in the investigated samples, and more importantly, the proportions of mica and smectite are negligible. Indeed, illite (%I > 95%) with a 1 M polytype structure and, to a minor extent, I-S (%I ~25–30%) with a 1M_d_ polytype structure dominate throughout the entire succession. Such a microstructure suggests an authigenic origin of these clay minerals (Baldermann et al. 2012, 2013) rather than reworking of detrital material from alluvial fan source areas (Tsagaan Ovoo Fm.) and subsequent deposition by dust and/or braided rivers (Hsanda Gol Fm. and Loh Fm.), as proposed by Höck et al. (1999) and Sun and Windley (2015).

More precisely, the Tsagaan Ovoo Fm. consists predominantly of quartz (41–50 wt%) and illite (33–46 wt%), in addition to moderate proportions of plagioclase, orthoclase (feldspar: 7–13 wt%) and hematite (2–10 wt%), with calcite, kaolinite, I-S, chlorite and Ti-oxides being minor constituents (<2 wt%). A similar mineralogical composition is evident for the Hsanda Gol Fm., except for the highly variable calcite content, attributed to calcrete horizons (1–69 wt%), and local restricted occurrences of zeolite, vermiculite and amphibole (each < 1 wt%) found between 32–43 m and 81–93 m adjacent to the weathered horizons of basalt groups I and II. Thus, illite (10–48 wt%), quartz (12–53 wt%), feldspar (3–13 wt%) and hematite ± goethite (1–9 wt%) are the main substituents, besides small admixtures of kaolinite, I-S, chlorite and halite, if present. The predominance of illite (17–45 wt%), quartz (18–46 wt%), calcite (2–59 wt%), feldspar (3–6 wt%) and Fe-(oxy)hydroxides (2–7 wt%) is also valid for the samples from the Loh Fm., which additionally contains minor amounts (<1 wt%) of kaolinite, I-S and chlorite as well as traces of zeolite, vermiculite and amphibole in close vicinity (94–101 m) to a basaltic horizon of group II (Fig. 3).

### Petrographic observations

**Fig. 4:**
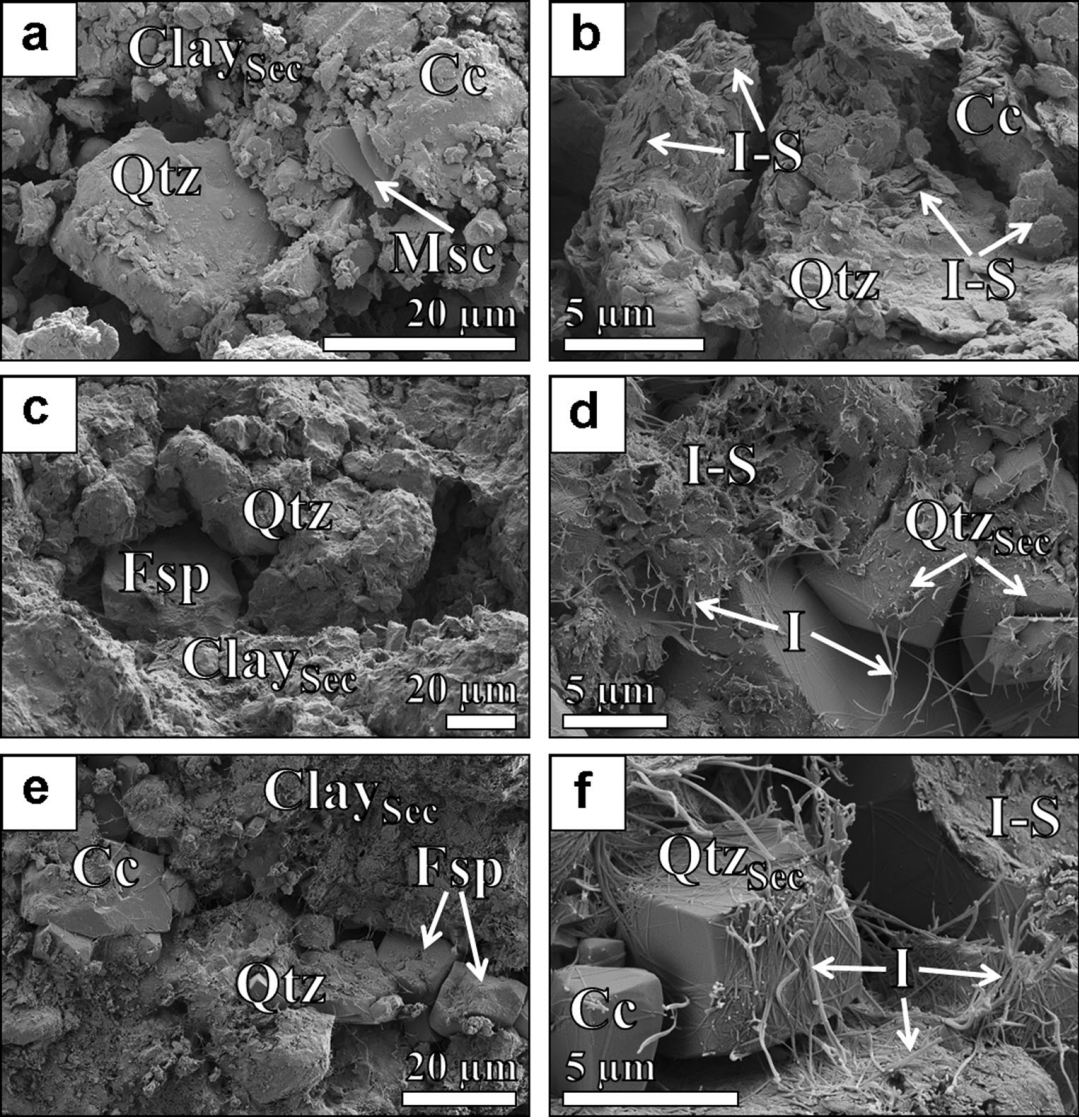
SEM-SE images of calcareous and illitized (arkosic) sandstones from the Oligocene Hsanda Gol Fm. collected at 78.5 m (**a**, **b**), 59.0 m (**c**, **d**) and 33.8 m (**e**, **f**). The association of hairy illite, platy I-S and euhedral quartz cement suggests intense alteration of the sediments at elevated temperatures. See text for further explanations. Abbreviations: *Qtz* detrital quartz, *Qtz*_*Sec*_ authigenic quartz cement, *Msc* muscovite, *Fsp* feldspar, *Cc* calcite, *Clay*_*Sec*_ secondary clay minerals, *I* hairy illite, *I-S* interstratified illite-smectite

Representative SEM images of rock chips from the Hsanda Gol Fm. collected at 78.5 m (Fig. 4a, b), 59.0 m (Fig. 4c, d) and 33.8 m (Fig. 4e, f) respectively show rock textures typical for immature (arkosic) sandstones that are weakly cemented with quartz and in particular with authigenic pore filling sheet silicates. Most of the quartz grains are diagenetically affected by the syntaxial (over)growth of quartz cement, and original quartz surfaces are not preserved. The clay minerals have either a lath-like morphology, with lath widths of 0.1 to 0.5 μm, <200 nm thickness and lengths ranging up to 20 μm (Fig. 4d, f) or a platy to irregular morphology with an average particle diameter of 2–4 μm and a particle thickness between 0.1 and 0.3 μm (Fig. 4b, d). EDS analyses of these two particle types reveal Si, Al and K as the major constituents, with the platy clays containing less K and Al and more Si and Na than the lath-like clays. In accordance with the mineralogical results, these particle morphologies and chemical compositions correspond either to “hairy” illite or interstratified I-S, respectively (Güven et al. 1980). The association of hairy 1 M illite, 1M_d_ I-S and quartz cement (Fig. 4d, f) is usually restricted to elevated temperatures around 70 to 150 °C and is therefore attributed to the advanced stages of sandstone diagenesis and the related burial diagenetic dissolution of feldspar-rich sediments (Nadeau et al. 1985; Lynch et al. 1997; Haszeldine et al. 2000; Baldermann et al. 2012). However, the shallow burial of the Valley of Lakes sediments (in the range of few hundred metres) is insufficient to create temperatures suitable for illitization (see discussion below).

**Fig. 5:**
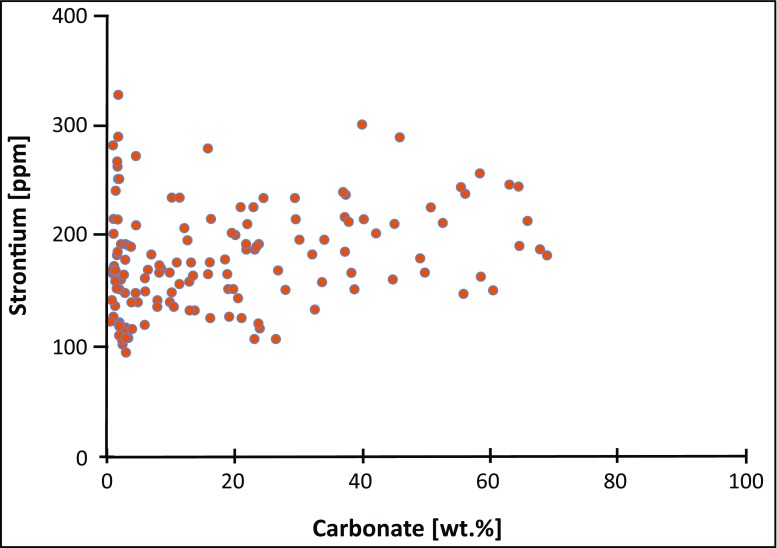
The Sr (ppm) vs. bulk rock CaCO_3_ [wt.%] cross plot points to precipitation of the soil carbonate (i.e. calcrete nodules and lenses) from meteoric solution

Calcrete occurs mainly as mm- to dm-sized honeycomb-type nodules and continuous hardplan in the Oligo-Miocene sediments, indicating an advanced stage of soil formation (stage III to IV, following the classification scheme of Goudie (1983) and Machette (1985)). The crypto- to microcrystalline nature of the calcite nodules and the absence of calcite spar and secondary dolomite suggest carbonate precipitation in the vadose zone rather than through interaction with groundwater (Quast et al. 2006). This observation is confirmed by both the presence of hematite (instead of goethite) in the carbonate and the comparable low Sr content (90 to 300 ppm) of the calcite, which is largely independent from the calcite content of the bulk rock samples (Fig. 5; Khadikikar et al. 2000). These features suggest that the pristine isotopic composition of the calcite is preserved in calcrete horizons and was virtually not disturbed by the later illitization (see below).

### Major, minor and trace element geochemistry

The element composition of samples from the Tsagaan Ovoo to Loh fms. is highly variable (Table 1) and follows mainly the changes in the ratio of the silicate relative to the carbonate fraction. Accordingly, the Al_2_O_3_ + MgO, SiO_2_ and CaO contents can be directly attributed to abundances of (sheet) silicates, such as illite and minor orthoclase, as well as quartz and calcite, respectively, complying with the petrographic and mineralogical results (Figs. 3 and 4). Na_2_O, Fe_2_O_3_ and TiO_2_ contents reflect variations in minor amounts of plagioclase, Fe-(oxy)hydroxides and Ti-oxides, i.e. rutile and anatase. MnO and P_2_O_5_ occur only in minor amounts.

**Table 1:** Major, minor and trace element composition of sediments from the Tsagaan Ovoo Fm., Hsanda Gol Fm. and Loh Fm. (Valley of Lakes, Central Mongolia) based on XRF data and percent in major minerals

Sample ID	Position (m)	Feldspat (wt%)	Illite (wt%)	Quartz (wt%)	Hematite (wt%)	Calcite (wt%)	SUM (wt%)	SiO_2_ (wt%)	Al_2_O_3_ (wt%)	Fe_2_O_3_ (wt%)	MnO (wt%)	MgO (wt%)	CaO (wt%)	Na_2_O (wt%)	K_2_O (wt%)	TiO_2_ (wt%)	P_2_O_5_ (wt%)	LOI (wt%)		SUM (wt%)		TIC (wt%)	
TAT/33b	96.11	9.4	36.2	40.6	5.3	8.6	100.00	55.43	15.61	6.37	0.14	2.61	3.68	1.52	2.89	0.74	0.11	10.90		100.00			
TAT/32d	94.71	5.8	15.4	11.7	2.6	64.5	100.00	17.31	4.85	1.91	0.05	1.14	37.58	0.42	0.86	0.23	0.06	35.60		100.00			
TAT/32b	93.66	8.2	36.9	40.7	6.1	8.2	100.00	55.38	15.09	6.08	0.11	2.26	5.00	1.25	2.67	0.74	0.12	11.32		100.00			
TAT/31	93.07	7.9	37.7	42.4	6.0	6.1	100.00	57.48	15.10	6.01	0.14	2.23	3.95	1.09	2.72	0.75	0.10	10.43		100.00			
TAT/30a	92.39	8.4	39.4	43.3	7.1	1.8	100.00	61.71	15.80	6.17	0.12	2.17	0.74	1.06	2.91	0.84	0.05	8.45		100.00			
TAT/29b	92.09	8.8	40.3	42.5	7.3	1.1	100.00	60.24	16.19	6.45	0.12	2.38	0.55	1.21	3.18	0.79	0.06	8.83		100.00			
TAT/28	91.22	6.7	26.7	25.3	4.1	37.3	100.00	36.79	9.63	3.79	0.08	1.67	22.17	0.75	1.82	0.48	0.20	22.64		100.00			
TAT/27b	91.09	8.2	37.8	38.8	6.9	8.3	100.00	56.21	14.78	5.91	0.15	2.18	5.34	1.01	2.80	0.74	0.13	10.76		100.00			
TAT/27a	90.76	7.8	15.2	16.8	1.7	58.4	100.00	22.11	5.29	2.04	0.04	1.18	34.40	0.92	0.98	0.28	0.11	32.65		100.00			
TAT/26	90.38	8.4	21.6	26.9	2.8	40.3	100.00	35.39	8.63	3.40	0.08	1.39	23.89	1.15	1.62	0.46	0.12	23.87		100.00			
TAT/25	90.01	10.0	36.5	44.3	7.0	2.3	100.00	60.64	15.39	6.05	0.11	2.12	1.27	1.35	2.90	0.83	0.06	9.28		100.00			
TAT/24a	89.85	7.3	17.9	17.3	2.1	55.5	100.00	24.95	6.54	2.57	0.06	1.48	31.36	1.07	1.28	0.31	0.10	30.29		100.00			
TAT/22c	87.94	10.4	37.5	34.5	5.4	12.2	100.00	51.85	13.75	5.48	0.15	2.33	8.05	1.52	2.83	0.67	0.07	13.31		100.00			
TAT/22a	86.54	12.9	33.5	38.6	4.7	10.2	100.00	54.10	14.77	5.92	0.16	2.35	4.64	2.57	3.20	0.70	0.07	11.53		100.00			
TAT/21a	86.24	11.2	47.9	32.8	7.0	1.1	100.00	58.10	16.51	6.46	0.09	2.35	0.53	1.62	3.86	0.74	0.08	9.68		100.00			
TAT/20b	85.94	10.1	38.5	41.9	6.7	2.7	100.00	57.66	16.07	6.76	0.20	2.52	0.77	1.12	2.82	0.77	0.12	11.19		100.00			
TAT/19a	84.92	9.5	40.6	42.6	6.3	1.0	100.00	59.13	16.20	6.40	0.15	2.42	0.56	1.54	2.98	0.78	0.06	9.78		100.00			
TAT/18a	83.87	8.4	24.0	18.2	3.5	45.9	100.00	30.51	7.92	3.11	0.06	1.72	26.59	1.28	1.51	0.38	0.05	26.87		100.00			
TAT/17b	83.24	8.0	24.6	26.4	4.0	37.0	100.00	36.63	9.54	3.76	0.09	2.00	21.23	1.22	1.88	0.46	0.06	23.13		100.00			
TAT/16a	82.11	8.7	29.0	37.9	4.2	20.2	100.00	49.82	13.18	5.25	0.13	2.23	10.45	1.19	2.68	0.64	0.06	14.37		100.00			
TAT/15c	81.84	10.8	32.7	33.0	4.0	19.6	100.00	46.69	12.37	4.93	0.11	2.28	12.14	1.58	2.55	0.60	0.06	16.70		100.00			
TAT/15a	80.67	9.9	35.8	33.8	4.2	16.3	100.00	48.98	13.16	5.22	0.11	2.18	10.55	1.34	2.82	0.63	0.23	14.79		100.00			
TAT/14b	80.09	12.5	36.9	34.8	4.4	11.4	100.00	51.23	13.74	5.49	0.13	2.28	7.75	1.90	2.86	0.67	0.09	13.88		100.00			
TAT/13	79.24	11.3	35.5	43.7	5.1	4.5	100.00	58.44	15.77	6.28	0.13	2.33	1.54	1.87	3.25	0.76	0.09	9.53		100.00			
TAT/12b	78.47	12.2	29.1	33.0	3.9	21.9	100.00	46.09	12.28	4.84	0.08	2.09	12.93	1.74	2.57	0.59	0.07	16.71		100.00			
TAT/11	69.86	10.1	23.2	29.2	3.4	34.1	100.00	41.43	10.99	4.32	0.09	1.91	17.41	1.48	2.33	0.54	0.13	19.37		100.00			
TAT/10	69.59	12.1	33.8	38.7	4.4	11.0	100.00	52.17	13.88	5.49	0.13	2.30	7.27	1.98	2.93	0.68	0.08	13.09		100.00			
TAT/9	69.22	13.1	30.8	31.2	3.8	21.0	100.00	45.55	12.08	4.77	0.11	2.09	12.87	2.23	2.55	0.59	0.22	16.95		100.00			
TAT/8b	68.66	12.4	37.7	42.9	4.8	2.3	100.00	59.00	15.38	6.07	0.12	2.66	1.64	2.13	3.40	0.76	0.08	8.77		100.00			
TAT/7b	67.99	12.9	36.9	37.8	4.5	7.9	100.00	54.98	14.38	5.71	0.12	2.39	5.20	2.20	3.22	0.71	0.18	10.92		100.00			
TAT/6b	67.24	11.5	25.4	30.1	3.5	29.5	100.00	43.75	11.43	4.50	0.09	2.06	14.70	2.15	2.53	0.56	0.30	17.93		100.00			
TAT/5	66.77	10.7	40.8	32.9	5.3	10.2	100.00	54.35	14.14	5.61	0.17	2.27	6.22	2.02	3.41	0.70	0.48	10.63		100.00			
TAT/4a	66.29	8.2	35.7	31.5	5.8	18.9	100.00	50.18	12.77	5.01	0.09	2.18	10.05	2.10	3.12	0.63	0.13	13.74		100.00			
TAT/3b	65.79	10.1	46.6	34.0	4.5	4.9	100.00	59.00	14.92	5.88	0.08	2.47	2.25	2.21	3.76	0.75	0.08	8.59		100.00			
TAT/2	65.08	10.6	39.5	30.6	5.4	13.8	100.00	53.66	13.70	5.40	0.07	2.23	7.43	1.67	3.38	0.67	0.13	11.66		100.00			
TAT/1b	64.47	9.6	31.5	32.3	4.8	21.8	100.00	47.04	11.88	4.82	0.08	2.23	13.22	1.42	2.64	0.58	0.09	16.00		100.00			
HTE/15	110.13	6.2	44.8	40.3	5.7	3.0	100.00	61.21	16.12	6.02	0.07	2.56	1.11	0.51	3.17	0.79	0.25	8.21		100.00		0.2	
HTE/14	109.78	5.8	43.8	43.0	5.5	2.0	100.00	61.04	16.02	6.28	0.17	2.63	1.11	0.44	3.12	0.78	0.17	8.25		100.00		0.3	
HTE/13b	108.98	5.4	41.2	43.4	6.1	4.0	100.00	61.30	16.19	6.23	0.10	2.93	1.12	0.30	3.07	0.79	0.21	7.76		100.00		0.3	
HTE/12	107.65	2.7	17.1	22.0	2.1	56.1	100.00	27.72	6.97	2.69	0.13	1.52	30.34	0.28	1.31	0.34	0.10	28.60		100.00		23.1	
HTE/11b	105.78	3.9	17.3	18.2	2.1	58.6	100.00	25.54	6.62	2.58	0.13	1.37	32.18	0.23	1.29	0.32	0.54	29.22		100.00		24.5	
HTE/11a	104.98	4.5	25.8	40.5	7.2	22.0	100.00	49.55	11.76	6.99	0.11	2.04	12.43	0.23	2.11	0.60	0.10	14.07		100.00		2.4	
HTE/9b	101.38	4.7	22.0	30.9	4.9	37.4	100.00	40.41	10.08	4.56	2.49	1.79	18.17	0.49	1.86	0.53	0.12	19.50		100.00		13.2	
HTE/9a	100.97	4.9	36.9	41.9	6.3	9.9	100.00	58.26	14.83	6.11	0.09	2.08	3.70	0.32	2.74	0.78	0.07	11.02		100.00		2.5	
HTE/8	100.40	6.0	38.2	46.2	2.6	7.0	100.00	66.63	11.90	3.93	0.07	1.23	3.98	0.76	3.53	0.64	0.15	7.20		100.00		2.8	
HTE/7b	100.00	4.0	23.5	24.0	3.4	45.1	100.00	32.36	8.07	2.97	0.29	1.41	26.70	0.34	1.48	0.41	0.08	25.91		100.00		20.8	
HTE/7a	98.73	5.0	34.9	45.6	6.6	8.0	100.00	61.68	15.47	6.13	0.06	2.12	3.11	0.35	2.75	0.81	0.06	7.46		100.00		2.0	
HTE/6	97.90	6.5	30.8	41.2	5.4	16.2	100.00	54.51	13.07	5.27	0.09	1.76	8.49	0.28	2.37	0.73	0.40	13.04		100.00		5.9	
HTE/5a	96.83	5.6	39.4	45.3	6.3	3.4	100.00	61.72	16.07	6.31	0.06	1.83	1.10	0.30	2.78	0.83	0.05	8.94		100.00		0.4	
HTE/4b	94.90	6.0	40.2	44.0	7.2	2.6	100.00	60.60	16.28	6.28	0.06	2.06	1.44	0.37	2.89	0.81	0.05	9.16		100.00		0.7	
HTE/3a	94.20	4.8	35.7	35.7	4.8	19.0	100.00	50.60	13.40	5.17	0.41	1.74	10.66	0.22	2.38	0.67	0.32	14.44		100.00		7.8	
HTE/2b	93.63	8.0	38.5	46.0	4.9	2.6	100.00	63.33	15.44	5.91	0.05	1.57	1.19	0.56	3.10	0.85	0.07	7.93		100.00		0.5	
HTE/2a	92.73	5.9	39.5	45.1	6.9	2.5	100.00	63.31	16.14	6.11	0.11	1.76	0.62	0.42	3.38	0.79	0.07	7.31		100.00		0.2	
HTE/1d	91.07	6.1	40.2	39.2	4.7	9.9	100.00	61.16	14.32	4.93	0.09	1.74	4.41	0.56	3.35	0.62	0.07	8.77		100.00		3.3	
HTE/1c	90.63	5.2	18.3	14.6	1.4	60.5	100.00	22.19	5.32	1.85	0.18	0.96	35.57	0.27	1.23	0.24	0.09	32.10		100.00		28.4	
HTE/1b	90.07	4.8	45.0	42.3	6.2	1.7	100.00	64.96	16.27	5.65	0.07	2.04	0.75	0.58	3.80	0.73	0.06	5.09		100.00		0.3	
HTE/1a	89.30	3.6	17.3	12.4	2.1	64.6	100.00	18.85	4.38	1.48	0.25	0.96	37.92	0.18	1.14	0.20	0.14	34.50		100.00		30.4	
TGR-C/20b	94.61	8.7	36.8	44.4	7.6	2.4	100.00	60.34	15.98	6.73	0.05	2.57	0.79	0.62	2.91	0.78	0.06	9.17		100.00			
TGR-C/20a	93.78	7.1	34.5	48.5	7.8	2.0	100.00	62.60	16.55	6.98	0.05	2.66	0.82	0.62	3.01	0.81	0.06	5.83		100.00			
TGR-C/19b	93.36	6.8	28.6	35.3	5.3	24.0	100.00	47.00	12.56	5.15	0.18	2.07	14.33	0.59	2.28	0.62	0.20	15.03		100.00			
TGR-C/19a	92.49	6.3	26.3	30.9	4.0	32.6	100.00	40.16	10.63	4.32	0.25	1.69	19.39	0.52	1.88	0.52	0.08	20.56		100.00			
TGR-C/18	91.84	6.7	27.3	33.7	4.3	28.0	100.00	45.19	11.85	4.79	0.18	1.81	15.07	0.58	2.06	0.60	0.18	17.70		100.00			
TGR-C/17a	91.18	5.9	28.3	38.8	6.4	20.6	100.00	50.84	12.92	5.19	0.16	1.76	10.86	0.48	2.16	0.66	0.06	14.91		100.00			
TGR-C/16b	90.49	8.2	34.6	47.8	7.3	2.0	100.00	62.08	15.74	6.28	0.07	1.86	1.04	0.64	2.47	0.83	0.04	8.96		100.00			
TGR-C/15	89.24	9.1	29.8	47.8	7.3	6.0	100.00	59.27	15.42	6.20	0.08	1.78	2.74	0.51	2.39	0.78	0.04	10.79		100.00			
TGR-C/14b trav	88.49	4.4	12.8	15.3	1.7	65.9	100.00	19.90	5.35	2.02	0.09	0.88	36.61	0.04	0.79	0.26	0.13	33.94		100.00			
TGR-C/14b	87.96	4.7	13.6	27.9	3.9	49.8	100.00	32.43	8.62	3.40	0.09	1.03	26.75	0.40	1.22	0.43	0.14	25.49		100.00			
TGR-C/13d	86.91	5.9	38.8	45.2	7.1	3.0	100.00	60.91	16.99	6.85	0.03	1.59	0.90	0.47	2.66	0.82	0.06	8.73		100.00			
TGR-C/13b	85.86	11.3	39.1	41.7	5.1	2.9	100.00	58.44	15.77	6.28	0.13	2.33	1.54	1.87	3.25	0.76	0.09	9.53		100.00			
TGR-C/12	84.77	12.2	29.7	30.0	4.9	23.2	100.00	46.09	12.28	4.84	0.08	2.09	12.93	1.74	2.57	0.59	0.07	16.71		100.00			
TGR-C/11b	84.69	8.2	36.4	45.5	6.9	3.0	100.00	60.43	16.61	6.49	0.15	1.75	1.47	0.89	2.82	0.79	0.05	8.56		100.00			
TGR-C/11a	84.32	12.1	28.2	25.1	4.4	30.2	100.00	41.43	10.99	4.32	0.09	1.91	17.41	1.48	2.33	0.54	0.13	19.37		100.00			
TGR-C/10b	84.14	6.3	10.4	13.0	2.4	67.9	100.00	16.79	4.42	1.68	0.07	1.01	38.65	0.53	0.78	0.21	0.07	35.79		100.00			
TGR-C/10a	83.24	12.1	35.6	33.7	5.4	13.2	100.00	52.17	13.88	5.49	0.13	2.30	7.27	1.98	2.93	0.68	0.08	13.09		100.00			
TGR-C/9	82.66	12.1	32.5	26.5	5.8	23.0	100.00	45.55	12.08	4.77	0.11	2.09	12.87	2.23	2.55	0.59	0.22	16.95		100.00			
TGR-C/8b	82.39	11.4	42.7	36.9	6.1	3.0	100.00	59.00	15.38	6.07	0.12	2.66	1.64	2.13	3.40	0.76	0.08	8.77		100.00			
TGR-C/8a	81.12	6.1	30.2	34.9	5.1	23.8	100.00	47.32	12.64	5.01	0.04	1.96	13.32	0.75	2.52	0.61	0.05	15.79		100.00			
TGR-C/7b	80.01	11.9	39.8	32.2	5.5	10.5	100.00	54.98	14.38	5.71	0.12	2.39	5.20	2.20	3.22	0.71	0.18	10.92		100.00			
TGR-C/6	78.97	12.5	32.3	26.1	4.5	24.6	100.00	43.75	11.43	4.50	0.09	2.06	14.70	2.15	2.53	0.56	0.30	17.93		100.00		2.0	
TGR-C/5	78.74	10.5	42.8	29.9	5.3	11.4	100.00	54.35	14.14	5.61	0.17	2.27	6.22	2.02	3.41	0.70	0.48	10.63		100.00		1.9	
TGR-C/4b	78.57	7.6	29.5	38.3	5.4	19.2	100.00	49.24	13.54	5.44	0.16	1.97	10.75	0.93	2.73	0.65	0.06	14.53		100.00		8.4	
TGR-C/4a	77.87	12.2	37.7	30.5	3.8	15.9	100.00	50.18	12.77	5.01	0.09	2.18	10.05	2.10	3.12	0.63	0.13	13.74		100.00		8.7	
TGR-C/3A	77.48	6.8	19.1	16.1	2.2	55.9	100.00	24.84	6.80	2.72	0.04	1.19	32.36	0.39	1.40	0.32	0.07	29.88		100.00		25.7	
TGR-C/3b	76.80	12.1	44.6	34.0	5.5	3.9	100.00	59.00	14.92	5.88	0.08	2.47	2.25	2.21	3.76	0.75	0.08	8.59		100.00			
TGR-C/2	75.47	9.6	40.5	31.6	5.2	13.0	100.00	53.66	13.70	5.40	0.07	2.23	7.43	1.67	3.38	0.67	0.13	11.66		100.00			
TGR-C/1	75.13	9.6	30.5	31.3	4.8	23.8	100.00	47.04	11.88	4.82	0.08	2.23	13.22	1.42	2.64	0.58	0.09	16.00		100.00		0.8	
SHG-D/30	91.20	5.3	18.9	22.4	2.5	50.8	100.00	29.82	7.89	3.06	0.32	1.35	28.91	0.41	1.38	0.37	0.05	26.46		100.00		3.4	
SHG-D/29a	90.82	6.3	31.2	41.0	5.7	15.9	100.00	54.92	14.30	5.59	0.24	2.52	7.30	0.77	2.43	0.65	0.04	11.23		100.00			
SHG-D/28b	90.45	7.3	26.2	32.1	4.8	29.6	100.00	46.65	11.81	4.58	0.20	2.03	15.20	0.65	2.05	0.57	0.04	16.21		100.00			
SHG-D/27b	90.05	6.9	37.4	46.0	6.3	3.4	100.00	62.41	16.17	6.38	0.19	2.25	1.72	0.72	2.91	0.78	0.07	6.42		100.00			
SHG-D/26b	73.05	6.4	22.5	28.0	3.1	40.0	100.00	36.48	9.41	3.67	0.26	1.64	23.48	0.41	1.81	0.46	0.04	22.34		100.00			
SHG-D/25	72.32	7.7	29.5	35.4	4.0	23.5	100.00	47.90	12.33	4.89	0.14	1.88	14.25	0.57	2.45	0.60	0.06	14.94		100.00			
SHG-D/24	71.98	5.9	36.7	39.1	5.6	12.7	100.00	55.19	14.31	5.66	0.10	2.24	6.98	0.73	2.84	0.69	0.07	11.18		100.00			
SHG-D/23j	71.85	5.9	24.5	28.1	4.2	37.3	100.00	39.69	10.29	4.02	0.12	1.72	20.88	0.48	2.08	0.49	0.09	20.15		100.00			
SHG-D/23i	71.38	5.3	23.7	24.9	3.9	42.2	100.00	34.99	9.06	3.57	0.16	1.54	24.74	0.41	1.82	0.44	0.06	23.21		100.00			
SHG-D/23 h	70.91	6.2	27.0	24.7	4.2	37.9	100.00	38.51	10.04	4.00	0.13	1.66	21.79	0.52	2.04	0.48	0.07	20.77		100.00			
SHG-D/23f	69.97	4.9	25.1	33.8	4.0	32.2	100.00	43.24	11.35	4.44	0.14	1.62	18.02	0.55	2.33	0.55	0.08	17.69		100.00			
SHG-D/23d	69.03	5.1	18.7	20.4	3.2	52.6	100.00	26.93	7.01	2.73	0.16	1.24	30.68	0.62	1.45	0.34	0.08	28.75		100.00			
SHG-D/23b	68.09	5.7	17.5	25.2	2.5	49.1	100.00	31.58	8.00	3.10	0.24	1.36	27.51	0.57	1.62	0.41	0.06	25.56		100.00			
SHG-D/23a	67.62	7.6	38.7	42.6	5.1	6.0	100.00	60.24	15.55	6.17	0.12	2.31	3.31	0.93	3.12	0.78	0.07	7.40		100.00			
SHG-D/22	67.05	4.7	16.0	14.7	1.5	63.1	100.00	19.21	4.92	1.88	0.09	1.20	37.02	0.34	1.02	0.24	0.11	33.97		100.00			
SHG-D/21	66.70	7.3	39.6	44.2	6.0	2.9	100.00	61.22	15.88	6.23	0.08	2.42	1.53	0.89	3.20	0.79	0.06	7.71		100.00			
SHG-D/20a	65.83	6.3	26.6	25.3	3.0	38.8	100.00	37.45	9.64	3.77	0.16	1.64	22.24	0.65	2.00	0.48	0.16	21.81		100.00			
SHG-D/19	65.42	9.0	34.3	44.5	5.7	6.5	100.00	59.31	15.12	5.94	0.23	2.39	2.88	1.10	3.18	0.76	0.09	9.01		100.00			
SHG-D/18	65.11	5.6	23.7	23.1	2.8	44.8	100.00	35.63	9.00	3.50	0.17	1.56	23.97	0.57	1.92	0.45	0.10	23.13		100.00			
SHG-D/17	64.93	7.8	39.4	44.8	5.9	2.0	100.00	61.49	15.60	6.14	0.07	2.41	1.59	0.96	3.28	0.79	0.11	7.57		100.00			
SHG-D/16b	64.30	8.6	26.7	27.8	3.1	33.7	100.00	41.09	10.23	3.97	0.17	1.67	19.99	0.81	2.20	0.53	0.14	19.22		100.00			
SHG-D/15	63.26	9.5	34.5	46.5	5.7	3.8	100.00	62.35	15.26	5.92	0.09	2.30	2.04	1.17	3.20	0.80	0.13	6.76		100.00			
SHG-D/14b	62.73	7.4	20.4	31.0	2.9	38.3	100.00	40.18	9.64	3.63	0.16	1.54	20.90	0.79	1.98	0.51	0.12	20.55		100.00			
SHG-D/13	61.98	12.7	30.3	48.3	4.1	4.6	100.00	64.05	14.17	5.11	0.07	1.98	1.96	1.55	2.81	0.78	0.16	7.35		100.00			
SHG-D/12	60.80	10.5	33.3	50.2	4.3	1.6	100.00	68.81	14.29	4.37	0.07	1.68	1.08	1.78	2.90	0.81	0.09	4.12		100.00			
SHG-D/11b	58.45	7.6	36.6	47.0	7.1	1.7	100.00	61.56	16.81	7.27	0.12	2.27	0.83	0.69	3.02	0.79	0.08	6.57		100.00			
SHG-D/10	57.28	9.8	37.7	41.6	9.0	1.8	100.00	57.72	16.92	10.23	0.25	2.52	0.74	0.59	3.21	0.77	0.11	6.94		100.00			
SHG-D/9d	57.23	9.2	39.4	44.4	6.1	1.0	100.00	61.04	16.46	6.32	1.45	2.39	0.80	0.76	3.18	0.80	0.07	6.75		100.00			
SHG-D/9c	56.53	8.6	40.5	43.5	6.3	1.2	100.00	62.72	16.62	6.55	0.07	2.45	0.58	0.81	3.38	0.82	0.06	5.95		100.00			
SHG-D/9b	55.83	10.3	39.7	43.6	5.5	0.9	100.00	66.04	15.02	5.70	0.06	2.28	0.48	1.50	3.22	0.79	0.06	4.84		100.00			
SHG-D/9a	55.13	9.6	32.5	52.6	3.5	1.8	100.00	69.13	12.69	4.43	0.03	2.08	0.91	1.79	2.55	0.76	0.04	5.61		100.00			
SHG-D/8	54.35	6.7	45.9	39.3	6.7	1.4	100.00	60.32	17.68	7.13	0.07	2.89	0.60	0.57	3.69	0.82	0.05	6.19		100.00			
SHG-D/7b	54.18	10.0	42.8	39.9	5.7	1.7	100.00	60.39	15.63	6.40	0.07	3.13	0.89	1.22	3.50	0.77	0.07	7.95		100.00			
SHG-D/6	51.43	2.8	12.5	13.8	1.9	69.0	100.00	18.42	5.22	2.14	0.08	1.10	37.44	0.22	1.07	0.24	0.59	33.48		100.00			
SHG-D/5	50.18	7.7	45.8	37.8	6.9	1.7	100.00	59.38	17.32	7.31	0.09	2.82	0.82	0.83	3.69	0.78	0.07	6.89		100.00			
SHG-D/4	49.35	10.2	47.5	35.0	7.1	1.4	101.00	58.70	17.04	7.55	0.10	2.96	0.64	1.00	3.93	0.72	0.08	7.30		100.00			
SHG-D/3a	47.55	9.8	40.8	42.4	5.6	1.4	100.00	62.51	16.25	6.63	0.07	2.83	0.32	1.20	3.77	0.77	0.11	5.54		100.00			
SHG-D/2	46.68	7.7	33.6	27.6	4.3	26.8	100.00	46.29	12.51	5.46	0.27	2.21	13.83	0.81	3.02	0.56	0.24	14.82		100.00			
SHG-D/1	45.76	12.0	41.3	40.0	5.7	1.1	100.00	61.65	15.30	6.00	0.07	3.29	0.57	2.08	3.45	0.74	0.27	6.57		100.00			
TGR-AB/35	40.46	11.9	37.5	40.4	5.6	4.6	100.00	61.62	13.44	6.70	0.02	2.82	1.89	1.46	3.01	0.62	0.48	7.94		100.00		0.2	
TGR-AB/34b	39.83	9.4	39.4	44.1	5.7	1.3	100.00	64.28	15.37	6.10	0.08	3.59	0.72	1.04	3.65	0.77	0.07	4.35		100.00		0.3	
TGR-AB/33b	38.36	8.4	29.0	31.6	4.5	26.5	100.00	45.52	11.53	4.54	0.09	2.39	16.32	0.78	2.69	0.57	0.10	15.47		100.00		12.6	
TGR-AB/32 l	37.63	6.7	34.0	33.5	4.6	21.1	100.00	52.43	13.46	5.32	0.10	2.67	10.17	0.95	3.24	0.66	0.21	10.79		100.00		7.9	
TGR-AB/32j	35.70	9.7	38.0	31.5	4.5	16.2	100.00	52.43	13.46	5.32	0.10	2.67	10.17	0.95	3.24	0.66	0.21	10.79		100.00		4.6	
TGR-AB/32i	34.73	9.4	36.2	36.1	5.3	12.9	100.00	56.72	14.11	5.57	0.11	2.45	6.62	1.03	3.35	0.70	0.13	9.22		100.00		5.6	
TGR-AB/32 h	33.76	8.2	32.7	31.6	4.4	23.2	100.00	48.82	12.31	4.80	0.07	2.57	13.16	0.76	2.94	0.57	0.12	13.90		100.00		10.1	
TGR-AB/32f	31.83	7.5	34.2	34.5	4.0	19.8	100.00	49.45	12.49	4.86	0.16	2.56	12.18	0.90	2.89	0.58	0.11	13.83		100.00		9.3	
TGR-AB/32d	29.90	9.4	38.2	29.4	4.5	18.5	100.00	49.90	12.60	4.70	0.38	2.35	11.28	1.40	3.17	0.59	0.21	13.43		100.00		8.5	
TGR-AB/32a	27.00	8.7	38.0	35.9	4.0	13.5	100.00	55.27	13.95	5.19	0.19	2.27	8.07	0.94	3.24	0.68	0.15	10.04		100.00		5.9	
TGR-AB/31	25.30	10.9	33.9	46.7	6.8	1.7	100.00	64.72	14.85	7.02	0.12	2.00	1.00	1.58	3.14	0.81	0.11	4.65		100.00		0.2	
TGR-AB/13	8.15	8.7	45.6	40.7	3.4	1.6	100.00	66.35	16.54	4.70	0.05	1.33	0.92	1.31	3.39	0.78	0.06	4.57		100.00		0.2	
TGR-AB/12	7.98	9.8	37.0	41.8	9.5	2.0	100.00	56.70	16.49	10.57	1.84	1.67	0.88	0.83	2.87	0.81	0.14	7.19		100.00		0.3	
TGR-AB/11a	6.82	7.1	39.4	50.3	2.4	0.8	100.00	70.75	13.67	3.77	0.04	1.41	0.47	1.54	3.65	0.52	0.04	4.15		100.00		0.2	
TGR-AB/9	3.46	13.2	33.2	43.2	9.8	0.6	100.00	59.27	16.80	10.96	0.05	1.19	0.31	1.65	3.04	0.74	0.16	5.82		100.00		0.2	
Sample ID	Position	Ba	Ce	Co	Cr	Cu	Ga	Hf	La	Nb	Ni	Pb	Rb	Sc	Sr	Th	U	V	Y	Zn	Zr	Th/Sc	Zr/Sc
	(m)	(ppm)	(ppm)	(ppm)	(ppm)	(ppm)	(ppm)	(ppm)	(ppm)	(ppm)	(ppm)	(ppm)	(ppm)	(ppm)	(ppm)	(ppm)	(ppm)	(ppm)	(ppm)	(ppm)	(ppm)		
TAT/33b	96.11	362	63	<20	79	40	18	<15	30	<20	45	31	129	20	171	15	<20	110	28	92	155	0.8	7.7
TAT/32d	94.71	151	20	<20	36	11	<10	<15	82	<20	<20	<20	38	30	246	14	<20	39	18	28	63	0.5	2.1
TAT/32b	93.66	399	63	<20	67	41	19	<15	62	<20	39	24	129	20	167	13	<20	124	31	95	164	0.7	8.2
TAT/31	93.07	395	88	<20	72	35	21	<15	47	23	46	31	130	20	150	12	<20	102	40	100	171	0.6	8.6
TAT/30a	92.39	424	71	<20	73	41	20	<15	31	<20	35	27	137	20	329	11	<20	100	22	110	186	0.6	9.3
TAT/29b	92.09	421	83	<20	70	44	21	<15	36	<20	44	30	137	20	216	10	<20	115	23	105	176	0.5	8.8
TAT/28	91.22	263	49	<20	44	25	12	<15	30	<20	20	21	81	23	218	9	20	83	20	63	114	0.4	5.1
TAT/27b	91.09	408	91	<20	63	38	20	<15	34	<20	43	31	126	20	174	8	<20	118	25	97	171	0.4	8.5
TAT/27a	90.76	163	30	<20	42	<20	<10	<15	94	<20	<20	<20	45	20	258	7	<20	42	15	40	101	0.4	5.2
TAT/26	90.38	204	50	<20	49	27	10	<15	30	<20	15	<20	73	28	216	6	<20	58	18	58	112	0.2	4.0
TAT/25	90.01	441	90	<20	76	38	20	<15	51	<20	33	<20	132	20	161	5	<20	101	25	101	196	0.3	9.8
TAT/24a	89.85	198	39	<20	43	<20	<10	<15	99	<20	21	<20	55	20	245	4	<20	52	15	44	86	0.2	4.3
TAT/22c	87.94	394	69	22	62	34	18	<15	37	<20	40	30	117	20	208	3	<20	104	24	87	149	0.2	7.5
TAT/22a	86.54	442	50	<20	64	39	20	<15	38	<20	43	30	127	20	149	2	<20	114	21	96	165	0.1	8.3
TAT/21a	86.24	740	76	<20	72	44	20	<15	62	<20	41	31	144	20	203	1	<20	96	25	102	170	0.1	8.5
TAT/20b	85.94	433	81	23	74	34	22	<15	74	<20	37	34	135	20	166	1	<20	131	23	99	177	0.1	8.8
TAT/19a	84.92	450	100	20	69	37	24	<15	53	<20	44	26	138	20	167	1	<20	120	25	92	183	0.1	9.2
TAT/18a	83.87	263	20	<20	52	22	10	<15	82	<20	22	<20	65	22	290	2	<20	67	30	48	119	0.1	5.5
TAT/17b	83.24	287	63	<20	45	27	10	<15	30	<20	24	21	82	21	241	3	<20	64	18	60	113	0.1	5.4
TAT/16a	82.11	400	62	<20	63	38	16	<15	30	<20	40	31	113	20	202	4	<20	97	25	81	149	0.2	7.3
TAT/15c	81.84	358	58	<20	61	32	17	<15	30	<20	33	23	106	20	203	5	<20	87	24	81	124	0.3	6.2
TAT/15a	80.67	364	78	<20	65	32	18	<15	30	<20	32	26	112	20	216	6	<20	91	35	86	152	0.3	7.6
TAT/14b	80.09	367	60	20	96	40	19	<15	63	<20	50	22	115	20	157	7	<20	98	25	84	148	0.4	7.4
TAT/13	79.24	479	88	<20	68	41	22	<15	68	<20	44	31	133	20	149	8	<20	109	25	98	161	0.4	8.0
TAT/12b	78.47	341	52	<20	53	33	16	<15	41	<20	29	20	103	20	188	9	<20	94	26	75	142	0.5	7.1
TAT/11	69.86	282	56	<20	51	29	12	<15	30	<20	29	<20	91	20	197	10	<20	92	21	70	136	0.5	6.8
TAT/10	69.59	424	93	<20	63	38	19	<15	30	<20	35	32	117	20	177	11	<20	104	24	87	172	0.6	8.6
TAT/9	69.22	356	76	<20	48	37	16	<15	30	<20	27	25	101	20	227	12	<20	99	23	78	133	0.6	6.6
TAT/8b	68.66	411	74	<20	67	44	20	<15	35	<20	39	25	135	20	193	13	<20	116	21	95	170	0.7	8.5
TAT/7b	67.99	380	64	<20	70	41	19	<15	44	<20	40	26	129	20	136	14	<20	119	26	92	160	0.7	8.0
TAT/6b	67.24	291	70	<20	53	35	14	<15	30	<20	28	23	98	20	235	15	<20	95	29	75	134	0.8	6.7
TAT/5	66.77	1119	69	<20	59	46	18	<15	54	<20	49	30	126	20	235	16	<20	115	31	98	157	0.8	7.9
TAT/4a	66.29	353	74	<20	59	37	18	<15	30	<20	28	23	116	20	166	17	<20	104	21	85	152	0.9	7.6
TAT/3b	65.79	649	75	<20	63	49	19	<15	30	<20	39	27	136	20	140	18	<20	125	22	99	175	0.9	8.8
TAT/2	65.08	347	72	<20	67	45	17	<15	40	<20	32	20	123	20	133	19	<20	116	25	89	158	1.0	7.9
TAT/1b	64.47	325	67	<20	108	77	16	<15	30	<20	46	21	103	20	193	20	<20	92	22	79	142	1.0	7.0
HTE/15	110.13	433	80	<20	74	33	20	<15	46	<20	50	21	128	20	118	20	<20	94	31	102	170	1.0	8.5
HTE/14	109.78	520	75	<20	72	38	24	<15	44	<20	50	32	126	20	122	20	<20	108	29	100	189	1.0	9.4
HTE/13b	108.98	469	89	<20	75	33	21	<15	44	20	50	20	126	20	116	20	<20	108	34	100	160	1.0	8.0
HTE/12	107.65	207	55	<20	41	<20	10	<15	86	<20	23	19.6	56	25.1	239	20	<20	59	16	42	88	0.8	3.5
HTE/11b	105.78	158	56	<20	49	<20	<10	<15	72	<20	29	<20	57	20	164	20	<20	33	27	42	70	1.0	3.5
HTE/11a	104.98	354	88	<20	52	34	13	<15	48	<20	32	31	97	20	211	20	<20	98	34	73	124	1.0	6.2
HTE/9b	101.38	1468	53	<20	59	34	12	<15	39	<20	27	<20	81	20	238	20	<20	123	18	68	125	1.0	6.2
HTE/9a	100.97	398	82	20	74	32	19	<15	46	<20	37	34	124	20	141	20	<20	103	22	83	170	1.0	8.5
HTE/8	100.40	606	88	<20	49	24	13	<15	64	<20	27	36	135	20	184	20	<20	71	17	53	146	1.0	7.3
HTE/7b	100.00	560	30	<20	51	<20	11	<15	63	<20	29	<20	68	27	212	20	<20	72	19	46	90	0.7	3.3
HTE/7a	98.73	391	83	24	83	44	20	<15	44	<20	42	26.5	126	21.1	142	20	<20	105	20	88	167	0.9	7.9
HTE/6	97.90	352	126	<20	63	26	20	<15	44	<20	33	30	110	20	177	20	<20	93	31	79	175	1.0	8.7
HTE/5a	96.83	369	88	<20	87	31	21	<15	43	<20	36	23	133	20	108	20	<20	100	23	97	187	1.0	9.3
HTE/4b	94.90	384	54	<20	78	33	24	<15	42	<20	38	28	139	20	109	20	<20	118	19	97	160	1.0	8.0
HTE/3a	94.20	600	79	<20	72	29	20	<15	30	<20	38	23	111	20	152	20	<20	96	22	81	144	1.0	7.2
HTE/2b	93.63	402	114	<20	74	33	18	<15	30	<20	38	32	148	20	114	20	<20	126	22	92	204	1.0	10.2
HTE/2a	92.73	446	65	21	67	31	22	<15	30	<20	53	25	149	20	102	20	<20	114	27	96	186	1.0	9.3
HTE/1d	91.07	453	79	<20	90	28	18	<15	38	<20	40	27	141	20	167	20	<20	91	22	75	158	1.0	7.9
HTE/1c	90.63	169	37	<20	44	<20	<10	<15	92	<20	<20	<20	51	24	151	20	<20	32	13	34	86	0.8	3.6
HTE/1b	90.07	505	83	<20	55	59	19	<15	44	<20	34	28.5	155	20	122	20	<20	89	19	102	182	1.0	9.1
HTE/1a	89.30	333	30	<20	34	<20	<10	<15	79	<20	<20	<20	45	25	192	20	<20	35	15	32	68	0.8	2.8
TGR-C/20b	94.61	332	59	<20	82	33	21	<15	56	<20	47	30	129	20	106	20	<20	106	26	90	189	1.0	9.5
TGR-C/20a	93.78	378	77	<20	79	38	23	<15	36	<20	53	34	133	20	110	20	<20	110	27	94	173	1.0	8.6
TGR-C/19b	93.36	332	77	<20	57	30	16	<15	34	<20	39	25	102	25	117	20	<20	90	31	71	152	0.8	6.0
TGR-C/19a	92.49	503	59	<20	54	27	15	<15	30	<20	29	<20	85	20	134	20	<20	78	26	58	141	1.0	7.2
TGR-C/18	91.84	338	49	<20	48	25	17	<15	32	<20	30	<20	95	20	152	20	<20	77	28	62	150	1.0	7.5
TGR-C/17a	91.18	407	91	<20	52	29	17	<15	30	<20	33	35	106	20	144	20	<20	70	23	67	185	1.0	9.2
TGR-C/16b	90.49	379	81	<20	81	37	23	<15	51	<20	34	31	129	20	119	20	<20	113	24	77	192	1.0	9.6
TGR-C/15	89.24	355	51	<20	74	26	21	<15	32	<20	29	31	124	20	120	20	<20	106	23	88	190	1.0	9.5
TGR-C/14b trav	88.49	240	51	<20	36	<20	<10	<15	101	22	<20	<20	48	26	214	20	<20	35	54	32	56	0.8	2.1
TGR-C/14b	87.96	263	71	<20	48	27	<10	<15	80	<20	27	26	72	30	167	20	<20	69	17	51	115	0.7	3.8
TGR-C/13d	86.91	329	82	<20	90	38	22	<15	55	<20	33	31	140	20	95	20	<20	120	21	95	180	1.0	9.0
TGR-C/13b	85.86	479	88	<20	68	41	22	<15	68	<20	44	31	133	20	149	8	<20	109	25	98	161	0.4	8.0
TGR-C/12	84.77	341	52	<20	53	33	16	<15	41	<20	29	20	103	20	188	9	<20	94	26	75	142	0.5	7.1
TGR-C/11b	84.69	578	76	23	78	37	24	<15	61	<20	45	26	140	20	108	20	<20	118	23	90	184	1.0	9.2
TGR-C/11a	84.32	282	56	<20	51	29	12	<15	30	<20	29	<20	91	20	197	10	<20	92	21	70	136	0.5	6.8
TGR-C/10b	84.14	204	30	<20	42	<20	<10	<15	92	<20	<20	<20	39	29	189	20	<20	33	15	29	61	0.7	2.1
TGR-C/10a	83.24	424	93	<20	63	38	19	<15	30	<20	35	32	117	20	177	11	<20	104	24	87	172	0.6	8.6
TGR-C/9	82.66	356	76	<20	48	37	16	<15	30	<20	27	25	101	20	227	12	<20	99	23	78	133	0.6	6.6
TGR-C/8b	82.39	411	74	<20	67	44	20	<15	35	<20	39	25	135	20	193	13	<20	116	21	95	170	0.7	8.5
TGR-C/8a	81.12	314	72	<20	70	30	18	<15	34	<20	29	25	109	24	121	20	<20	93	23	81	152	0.8	6.3
TGR-C/7b	80.01	380	64	<20	70	41	19	<15	44	<20	40	26	129	20	136	14	<20	119	26	92	160	0.7	8.0
TGR-C/6	78.97	291	70	<20	53	35	14	<15	30	<20	28	23	98	20	235	15	<20	95	29	75	134	0.8	6.7
TGR-C/5	78.74	1119	69	<20	59	46	18	<15	54	<20	49	30	126	20	235	16	<20	115	31	98	157	0.8	7.9
TGR-C/4b	78.57	578	72	20	68	36	16	<15	30	<20	38	37	121	20	127	20	<20	103	28	90	171	1.0	8.5
TGR-C/4a	77.87	353	74	<20	59	37	18	<15	30	<20	28	23	116	20	166	17	<20	104	21	85	152	0.9	7.6
TGR-C/3A	77.48	171	33	<20	49	<20	<10	<15	77	<20	20	22	61	20	148	20	<20	44	19	46	103	1.0	5.1
TGR-C/3b	76.80	649	75	<20	63	49	19	<15	30	<20	39	27	136	20	140	18	<20	125	22	99	175	0.9	8.8
TGR-C/2	75.47	347	72	<20	67	45	17	<15	40	<20	32	20	123	20	133	19	<20	116	25	89	158	1.0	7.9
TGR-C/1	75.13	325	67	<20	108	77	16	<15	30	<20	46	21	103	20	193	20	<20	92	22	79	142	1.0	7.0
SHG-D/30	91.20	732	47	<20	50	<20	12	<15	72	<20	27	<20	67	25	227	20	<20	93	25	46	88	0.8	3.5
SHG-D/29a	90.82	609	72	<20	58	36	17	<15	30	<20	46	26	122	20	280	20	<20	137	22	77	155	1.0	7.7
SHG-D/28b	90.45	576	39	<20	46	30	17	<15	30	<20	27	22	97	20	216	20	<20	110	21	64	150	1.0	7.5
SHG-D/27b	90.05	558	81	<20	70	36	23	<15	30	<20	42	33	136	20	192	20	<20	143	24	92	171	1.0	8.6
SHG-D/26b	73.05	547	56	22	45	25	13	<15	30	<20	35	26	81	20	302	20	<20	75	32	59	112	1.0	5.6
SHG-D/25	72.32	444	51	<20	54	35	14	<15	30	<20	32	22	104	20	191	20	<20	101	25	80	131	1.0	6.5
SHG-D/24	71.98	437	60	<20	57	42	16	<15	30	<20	43	29	121	20	197	20	<20	113	23	89	167	1.0	8.3
SHG-D/23j	71.85	386	45	<20	55	29	12	<15	30	<20	29	26	87	20	186	20	<20	88	24	67	136	1.0	6.8
SHG-D/23i	71.38	395	61	<20	58	27	10	<15	30	<20	28	<20	79	20	203	20	<20	71	17	59	104	1.0	5.2
SHG-D/23 h	70.91	399	46	<20	142	22	14	<15	30	<20	53	<20	83	20	214	20	<20	85	24	63	105	1.0	5.3
SHG-D/23f	69.97	432	68	<20	62	30	14	<15	30	<20	24	23	95	20	184	20	<20	79	25	74	107	1.0	5.4
SHG-D/23d	69.03	338	46	<20	54	<20	10	<15	97	<20	28	<20	60	20	213	20	<20	49	18	50	97	1.0	4.8
SHG-D/23b	68.09	475	44	<20	53	27	<10	<15	66	<20	28	22	68	21	180	20	<20	71	22	52	96	1.0	4.7
SHG-D/23a	67.62	483	82	<20	73	37	18	<15	29	<20	41	31	134	20	162	20	<20	124	23	92	173	1.0	8.7
SHG-D/22	67.05	196	33	<20	38	<20	<10	<15	80	<20	21	<20	42	31	247	20	<20	31	16	38	77	0.7	2.5
SHG-D/21	66.70	484	78	<20	93	39	21	<15	41	<20	50	32	135	20	179	20	<20	120	24	99	173	1.0	8.6
SHG-D/20a	65.83	424	55	<20	48	24	15	<15	30	<20	27	23	84	23	152	20	<20	72	19	61	109	0.9	4.8
SHG-D/19	65.42	652	96	31	68	43	21	<15	68	<20	44	30	131	20	170	20	<20	122	22	96	165	1.0	8.3
SHG-D/18	65.11	473	37	<20	47	27	12	<15	30	<20	27	<20	79	24	161	20	<20	77	19	56	95	0.8	4.0
SHG-D/17	64.93	463	64	<20	72	37	20	<15	45	<20	40	31	135	20	152	20	<20	114	25	97	184	1.0	9.2
SHG-D/16b	64.30	472	69	<20	52	25	13	<15	30	<20	28	<20	90	20	159	20	<20	83	21	63	134	1.0	6.7
SHG-D/15	63.26	463	88	<20	73	31	17	<15	55	<20	39	32	135	20	191	20	<20	112	28	95	174	1.0	8.7
SHG-D/14b	62.73	425	65	<20	45	25	13	<15	31	<20	24	23	86	20	167	20	<20	67	29	61	130	1.0	6.5
SHG-D/13	61.98	423	86	<20	54	34	19	<15	74	21	32	28	133	20	210	23	<20	89	34	85	205	1.1	10.3
SHG-D/12	60.80	475	103	<20	55	28	17	<15	60	<20	25	26	138	20	183	20	<20	65	31	76	249	1.0	12.4
SHG-D/11b	58.45	481	101	21	82	40	23	<15	39	21	43	30	155	20	264	23	<20	149	32	113	156	1.1	7.8
SHG-D/10	57.28	636	59	26	88	45	24	<15	47	<20	46	28	157	20	252	21	<20	200	25	119	143	1.1	7.1
SHG-D/9d	57.23	2439	99	<20	68	39	21	<15	65	22	72	27	150	20	283	20	<20	139	37	107	170	1.0	8.5
SHG-D/9c	56.53	414	81	<20	76	32	23	<15	53	<20	35	<20	151	20	173	20	<20	109	27	103	176	1.0	8.8
SHG-D/9b	55.83	435	81	<20	67	28	22	<15	59	<20	40	41	134	20	170	20	<20	89	30	90	189	1.0	9.4
SHG-D/9a	55.13	332	88	<20	60	31	15	<15	61	<20	31	<20	110	20	291	20	<20	72	28	69	273	1.0	13.6
SHG-D/8	54.35	456	56	22	87	43	22	<15	64	<20	49	36	159	20	242	20	<20	122	27	115	171	1.0	8.5
SHG-D/7b	54.18	444	100	<20	69	40	21	<15	59	<20	49	26	136	20	268	20	<20	108	30	102	164	1.0	8.2
SHG-D/6	51.43	185	51	<20	46	<20	<10	<15	106	26	<20	<20	49	22	183	20	<20	39	65	36	71	0.9	3.2
SHG-D/5	50.18	496	71	<20	80	38	22	<15	49	<20	45	37	159	20	216	20	<20	120	33	114	133	1.0	6.7
SHG-D/4	49.35	503	100	<20	76	34	23	<15	45	<20	43	<20	151	20	160	20	<20	122	25	109	153	1.0	7.7
SHG-D/3a	47.55	484	68	<20	82	38	20	<15	30	<20	47	20	141	20	169	20	<20	109	29	104	184	1.0	9.2
SHG-D/2	46.68	693	51	<20	71	29	17	<15	32	<20	40	24	110	20	169	20	<20	97	24	84	152	1.0	7.6
SHG-D/1	45.76	465	83	<20	67	36	19	<15	48	20	42	26	133	20	127	20	<20	117	35	98	199	1.0	9.9
TGR-AB/35	40.46	492	49	24	75	66	24	<15	30	26	72	25	79	20	273	20	<20	56	13	63	165	1.0	8.2
TGR-AB/34b	39.83	445	70	21	67	72	21	<15	44	<20	54	31	155	20	137	20	<20	85	26	111	171	1.0	8.5
TGR-AB/33b	38.36	310	85	<20	63	28	14	<15	30	<20	31	27	117	20	107	20	<20	75	24	72	144	1.0	7.2
TGR-AB/32 l	37.63	380	91	<20	65	32	15	<15	30	<20	34	22	130	20	126	20	<20	89	25	89	176	1.0	8.8
TGR-AB/32j	35.70	380	91	<20	65	32	15	<15	30	<20	34	22	130	20	126	20	<20	89	25	89	176	1.0	8.8
TGR-AB/32i	34.73	424	86	<20	62	27	18	<15	57	<20	34	31	135	20	159	20	<20	107	28	93	160	1.0	8.0
TGR-AB/32 h	33.76	286	63	<20	61	30	16	<15	30	<20	30	<20	119	22	107	20	<20	89	23	79	135	0.9	6.1
TGR-AB/32f	31.83	400	68	<20	61	32	13	<15	49	<20	31	24	119	20	152	20	<20	85	23	78	173	1.0	8.6
TGR-AB/32d	29.90	854	61	<20	61	30	16	<15	30	<20	35	23	125	20	179	20	<20	82	28	82	156	1.0	7.8
TGR-AB/32a	27.00	532	99	<20	61	25	18	<15	49	<20	32	40	144	20	165	21	<20	83	25	84	169	1.1	8.5
TGR-AB/31	25.30	441	93	<20	75	32	20	<15	53	<20	37	25	141	20	186	22	<20	115	25	92	270	1.1	13.5
TGR-AB/13	8.15	495	98	<20	59	22	21	<15	67	<20	<20	20	159	20	153	24	<20	58	28	66	316	1.2	15.8
TGR-AB/12	7.98	4111	62	46	72	40	23	<15	47	<20	50	24	151	20	252	31	<20	250	27	95	230	1.5	11.5
TGR-AB/11a	6.82	481	54	<20	32	<20	16	<15	31	<20	<20	20	145	20	142	20	<20	47	18	51	227	1.0	11.3
TGR-AB/9	3.46	481	62	28	83	46	24	<15	30	<20	64	37	139	20	123	27	<20	160	20	76	274	1.4	13.7

**Fig. 6:**
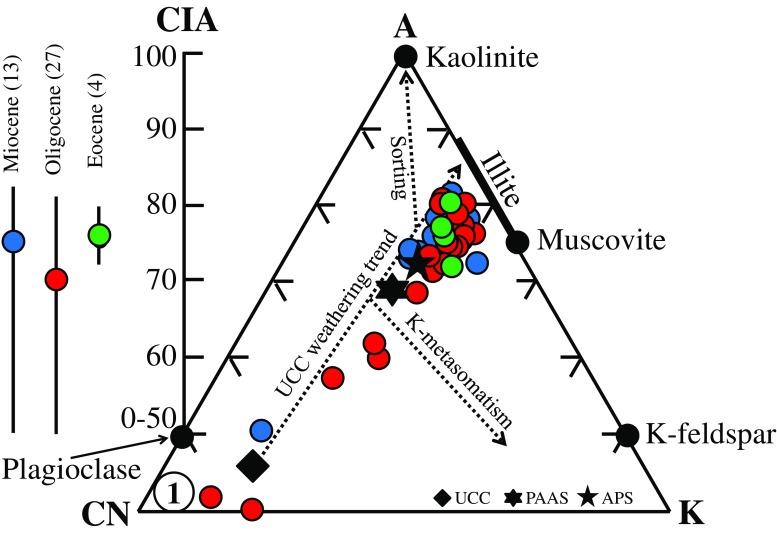
A-CN-K (Al_2_O_3_-CaO* + Na_2_O-K_2_O) diagram of Nesbitt and Young (1984) showing the chemical compositions of intensively weathered (calcareous and illitized) sediments from the Tsagaan Ovoo Fm., Hsanda Gol Fm. and Loh Fm. (Valley of Lakes, Central Mongolia). Compositions of Upper Continental Crust (UCC), Average Proterozoic Shale (APS), Post-Archean average Australian Shale (PAAS) and basalt protolith (1) are included for comparison (Bahlburg and Dobrzinski 2011 and references herein). Note that the lower part of the diagram (A < 40) is not shown. The range and averages (*dots*) of the CIA values are indicated in the left part of the figure

The averaged CIA values (left part of Fig. 6) ranged between 70 and 76 and plot slightly below the field of illite (75–90). This indicates that the Tsagaan Ovoo to Loh fms. sediments underwent sustained chemical weathering, considering that fresh basalt, unweathered granite and granodiorite and feldspar have CIA values of 30–45, 45–55 and 50, respectively (Bahlburg and Dobrzinski 2011). Moreover, by transferring the XRF data in Nesbitt and Young`s (1984) A-CN-K (Al_2_O_3_-CaO* + Na_2_O-K_2_O) diagram (Fig. 6), it becomes clear that the majority of the samples plot either near the compositional range of Post-Archean average Australian Shale (PAAS) and Average Proterozoic Shale (APS) or follow the predicted weathering trend for basalt protolith and Upper Continental Crust (UCC) rocks (von Eynatten 2004; Bahlburg and Dobrzinski 2011 and references herein). This observation demonstrates that sorting, K-metasomatism and frictional variations in palaeo-climate and tectonic setting played only a minor role in affecting the chemical composition of the sediments and basalt groups I to II, in contrast to the pronounced illitization event(s) (Fedo et al. 1995; Armstrong-Altrin et al. 2004; Yang et al. 2004).

The positive correlation of large-ion lithophile elements such as K, Rb and Ba (also-called LILE) as well as Cr, Zn and V with Al_2_O_3_ suggests their incorporation in sheet silicates, i.e. illite and I-S, and/or sorption onto clay mineral surfaces (Table 1). High K_2_O/Rb ratios, in the range from 165 to 384, point to moderate-to-high weathering intensities and arid to semiarid climatic conditions (Jin et al. 2006), a feature confirmed by the high CIA values. The low but highly variable Sr contents (95–330 ppm; Fig. 5) roughly correspond to variations in the fraction of calcite present in calcrete horizons (Table 1). The patterns of Co, Cu, Ga, Hf, Nb, Ni, Pb and U are mostly inconspicuous. However, positive trends of the heavy rare earth element (HREE) Y, with incompatible TiO_2_ and the immobile high field strength elements (HFSE) Ce, La, Sc, (Th) and Zr (Table 1), point to their presence in heavy minerals that have been delivered from metamorphosed and magmatic source rocks exposed in the hinterland (McLennan et al. 1993).

### Carbon and oxygen isotopes

The δ^18^O values of carbonates present in calcrete horizons in the lower part of the Hsanda Gol Fm., i.e. below the basalt I group, reveal the heaviest isotopic signatures throughout the entire investigated succession and very high variation in the range from −0.2 to −9.3‰ (see Fig. 2 and Table 2). A significantly lower scatter in the δ^18^O values, from −7.0 to −9.6‰, is evident above the basalt I group in the Hsanda Gol Fm. In the Loh Fm. at the Hotuliin Teeg section, the δ^18^O values show again higher variability with values from −12.9 to −8.6‰. Overall, an evolution towards progressively lighter δ^18^O values of the carbonate is evident up-section in the profile, starting in the upper biozone B.

**Table 2:** δ^13^C and δ^18^O isotope composition of sediments from the Tsagaan Ovoo Fm., Hsanda Gol Fm. and Loh Fm. (Valley of Lakes, Central Mongolia)

BIOZONE	High in profile (m)	SAMPLE	δ^13^C_carb, V-PDB_	δ^18^O_carb, V-PDB_
Biozone D	108.42	HTE/13a carb	−8.1	−10.2
Biozone D	107.65	HTE/12 carb	−4.4	−8.6
Biozone D	105.78	HTE/11b carb	−5.4	−8.9
Biozone D	104.98	HTE/11a carb	−7.3	−11.0
Biozone D	101.38	HTE/9b carb	−5.1	−9.5
Biozone D	100.97	HTE/9a carb	−6.3	−11.2
Biozone D	100.40	HTE/8 carb	−6.1	−10.6
Biozone D	100.00	HTE/7b carb	−5.7	−8.9
Biozone D	98.73	HTE/7a carb	−6.4	−11.7
Biozone C1-D	97.90	HTE/6 carb	−6.3	−10.3
Biozone C1-D	97.50	HTE/5b carb	−8.9	−10.2
Biozone C1-D	94.90	HTE/4b carb	−9.0	−12.9
Biozone C1-D	94.43	HTE/4a carb	−6.3	−9.6
Biozone C1-D	94.20	HTE/3A carb	−9.4	−11.0
Biozone C1-D	94.00	HTE/3 carb	−8.8	−11.0
Biozone C1-D	93.36	TGR-C/19b	−3.8	−8.7
Biozone C1-D	92.49	TGR-C/19a	−4.8	−8.5
Biozone C1	91.84	TGR-C/18	−4.7	−7.8
Biozone C1	91.41	TGR-C/17b	−5.0	−8.6
Biozone C1	91.18	TGR-C/17a	−4.5	−8.3
Biozone C1	91.06	TGR-C/16c	−4.9	−8.5
Biozone C1	89.24	TGR-C/15	−6.1	−7.8
Biozone C1	88.49	TGR-C/14b	−6.1	−9.1
Biozone C1	85.86	TGR-C/13b	−5.7	−9.1
Biozone C1	84.77	TGR-C/12	−5.4	−7.7
Biozone C1	84.32	TGR-C/11a	−6.8	−9.1
Biozone C1	84.14	TGR-C/10b	−5.9	−9.6
Biozone C	83.24	TGR-C/10a	−5.7	−7.8
Biozone C	82.66	TGR-C/9	−5.8	−8.8
Biozone C	82.39	TGR-C/8b	−5.6	−7.6
Biozone C	81.12	TGR-C/8a	−5.8	−8.3
Biozone C	80.49	TGR-C/7c	−5.5	−9.1
Biozone C	80.01	TGR-C/7b	−5.4	−9.3
Biozone C	79.52	TGR-C/7a	−5.9	−7.6
Biozone C	78.97	TGR-C/6	−5.9	−9.2
Biozone C	78.74	TGR-C/5	−5.4	−9.2
Biozone C	78.57	TGR-C/4b	−5.6	−8.1
Biozone C	77.87	TGR-C/4a	−5.6	−8.7
Biozone C	77.48	TGR-C/3A	−6.1	−8.8
Biozone C	77.40	TGR-C/3c	−5.1	−7.0
Biozone C	76.80	TGR-C/3b	−5.5	−8.8
Biozone C	76.20	TGR-C/3a	−5.3	−8.6
Biozone C	75.47	TGR-C/2	−5.9	−8.6
Biozone C	75.13	TGR-C/1	−5.9	−9.2
Biozone B	72.32	SHG-D/25	−5.4	−8.8
Biozone B	71.98	SHG-D/24	−5.2	−9.3
Biozone B	71.85	SHG-D/23j	−5.5	−8.5
Biozone B	71.38	SHG-D/23i	−5.3	−8.3
Biozone B	70.91	SHG-D/23 h	−5.4	−8.5
Biozone B	70.44	SHG-D/23 g	−5.0	−8.2
Biozone B	69.97	SHG-D/23f	−5.2	−8.3
Biozone B	69.50	SHG-D/23e	−5.3	−7.9
Biozone B	69.03	SHG-D/23d	−5.4	−8.1
Biozone B	68.56	SHG-D/23c	−4.6	−7.8
Biozone B	68.09	SHG-D/23b	−5.4	−8.3
Biozone B	67.62	SHG-D/23a	−5.0	−8.9
Biozone B	67.05	SHG-D/22	−5.2	−8.8
Biozone B	66.70	SHG-D/21	−5.1	−9.1
Biozone B	66.40	SHG-D/20b	−5.5	−8.3
Biozone B	65.83	SHG-D/20a	−5.8	−7.9
Biozone B	65.42	SHG-D/19	−4.9	−8.1
Biozone B	65.11	SHG-D/18	−5.3	−8.1
Biozone B	64.93	SHG-D/17	−5.1	−9.2
Biozone B	64.73	SHG-D/16c	−5.6	−7.8
Biozone B	64.30	SHG-D/16b	−5.6	−8.3
Biozone B	63.86	SHG-D/16a	−5.5	−7.9
Biozone B	63.26	SHG-D/15	−5.1	−8.6
Biozone B	62.73	SHG-D/14b	−5.9	−8.0
Biozone B	62.43	SHG-D/14a	−5.6	−8.6
Biozone B	61.98	SHG-D/13	−5.3	−7.6
Biozone B	51.43	SHG-D/6	−7.3	−8.5
Biozone B	46.68	SHG-D/2	−7.6	−8.7
Biozone A	38.73	TGR-AB/33c	−9.2	−9.1
Biozone A	38.36	TGR-AB/33b	−8.3	−8.6
Biozone A	38.00	TGR-AB/33a	−7.2	−9.3
Biozone A	37.63	TGR-AB/32 l	−7.5	−8.4
Biozone A	36.66	TGR-AB/32 k	−7.0	−8.9
Biozone A	35.70	TGR-AB/32j	−7.0	−4.6
Biozone A	34.73	TGR-AB/32i	−6.8	−1.5
Biozone A	33.76	TGR-AB/32 h	−6.9	−7.2
Biozone A	32.80	TGR-AB/32 g	−6.6	−8.6
Biozone A	31.83	TGR-AB/32f	−6.8	−2.6
Biozone A	30.86	TGR-AB/32e	−6.8	−0.2
Biozone A	29.90	TGR-AB/32d	−6.7	−5.8
Biozone A	28.93	TGR-AB/32c	−6.4	−8.4
Biozone A	27.96	TGR-AB/32b	−7.2	−6.1
Biozone A	27.00	TGR-AB/32a	−7.0	−6.9
Biozone C1	91.20	SHG-D/30	−5.5	−8.1
Biozone C1	91.00	SHG-D/29b	−5.7	−8.2
Biozone C1	90.82	SHG-D/29a	−5.4	−8.8
Biozone C1	90.18	SHG-D/28a	−5.4	−8.4
Biozone C1	89.55	SHG-D/26b	−5.6	−8.8
Biozone C1	89.15	SHG-D/26a	−5.5	−10.3
Biozone C1-D	96.11	TAT/33b	−5.3	−9.6
Biozone C1-D	95.18	TAT/33a	−4.9	−9.7
Biozone C1-D	94.71	TAT/32d	−4.7	−8.8
Biozone C1-D	94.19	TAT/32c	−5.1	−9.6
Biozone C1-D	93.66	TAT/32b	−5.4	−9.5
Biozone C1-D	93.14	TAT/32a	−5.1	−9.0
Biozone C1-D	93.07	TAT/31	−5.4	−8.9
Biozone C1-D	92.99	TAT/30b	−5.6	−9.4
Biozone C1	91.22	TAT/28	−4.9	−9.0
Biozone C1	91.09	TAT/27b	−5.4	−9.5
Biozone C1	90.76	TAT/27a	−5.2	−9.3
Biozone C1	90.01	TAT/25	−5.0	−8.4
Biozone C1	88.81	TAT/23	−5.2	−9.0
Biozone C1	88.64	TAT/22d	−5.2	−9.3
Biozone C1	87.94	TAT/22c	−4.9	−9.2
Biozone C1	87.24	TAT/22b	−4.9	−8.9
Biozone C1	86.54	TAT/22a	−4.9	−9.2
Biozone C1	83.87	TAT/18a	−5.0	−9.3
Biozone C	83.54	TAT/17c	−4.7	−8.8
Biozone C	83.24	TAT/17b	−4.8	−9.0
Biozone C	82.94	TAT/17a	−4.8	−9.2
Biozone C	82.64	TAT/16b	−4.8	−9.7
Biozone C	82.11	TAT/16a	−4.9	−9.6
Biozone C	81.84	TAT/15c	−4.8	−9.2
Biozone C	81.26	TAT/15b	−4.2	−8.6
Biozone C	80.67	TAT/15a	−5.5	−9.7
Biozone C	80.09	TAT/14b	−4.9	−9.2
Biozone C	79.59	TAT/14a	−4.9	−9.0
Biozone C	79.04	TAT/12c	−4.8	−8.8
Biozone C	78.47	TAT/12b	−4.8	−8.6
Biozone C	77.91	TAT/12a	−4.8	−8.9
Biozone B	68.99	TAT/8c	−4.7	−8.7
Biozone B	68.32	TAT/8a	−4.5	−8.9
Biozone B	67.99	TAT/7b	−4.7	−8.8
Biozone B	67.62	TAT/7a	−4.6	−8.7
Biozone B	67.44	TAT/6c	−4.5	−8.8
Biozone B	67.24	TAT/6b	−4.6	−8.8
Biozone B	67.04	TAT/6a	−4.6	−8.9
Biozone B	66.77	TAT/5	−4.6	−8.7
Biozone B	66.62	TAT/4b	−4.6	−8.7
Biozone B	66.29	TAT/4a	−4.7	−9.1
Biozone B	66.12	TAT/3c	−4.8	−9.4
Biozone B	65.79	TAT/3b	−4.5	−8.3
Biozone B	65.08	TAT/2	−4.8	−8.8
Biozone B	65.00	TAT/1c	−4.7	−8.7
Biozone B	64.47	TAT/1b	−4.9	−9.3
Biozone B	63.93	TAT/1a	−4.8	−9.1

Below 55 m in the profile (in the lower part of the Hsanda Gol Fm.), the δ^13^C values vary from −6.4 to −7.6‰, except for the two samples taken immediately below basalt I, which yielded −9.2 and −8.3‰ of δ^13^C, respectively. The remaining sections of the Hsanda Gol Fm. display heavier δ^13^C values, ranging from −6.8 to −3.8‰. Only two outliers were recognised, with δ^13^C value of approximately −7.2‰ (TAT 24 and TGR-C 11). Finally, the Loh Fm. is characterised, as for the δ^18^O values, by a stronger variability in the δ^13^C values in the range from −9.4 to −4.4‰.

## Discussion

Based on a seminal sedimentological, petrographic and chemical description of the Eocene to Miocene sedimentary succession and embedded basalt groups I to III, Höck et al. (1999) discussed the provenance of the sediments from the Valley of Lakes (Mongolia) and proposed a partly aeolian origin for the finest fraction of the Hsanda Gol to Loh fms. Their hypothesis was mainly based on a general coincidence between the grain size distribution patterns of the Valley of Lakes sediments compared with those of modern Loess deposits from Kansas. However, Höck et al. (1999) noticed some discrepancies in the grain size distribution patterns of sediments from the Hsanda Gol and Loh fms. and interpreted them to be attributed either to short distance aeolian transport or coupled aeolian–fluvial transport mechanisms. Sun and Windley (2015) proposed an aeolian origin of the fine fraction based on REE patterns and comparison of grain size distribution patterns with that from the modern Chinese Loess Plateau. They concluded that the finest fraction of the Hsanda Gol and Loh fms. was deposited along with the Cenozoic cooling through long distance transport.

The herein obtained mineralogical and petrographic results (Figs. 3 and 4), however, clearly reveal a diagenetic origin of illite, which represents a main constituent of the fine fraction of the Hsanda Gol and Loh fms. Moreover, the association of hairy 1 M illite, flaky 1M_d_ I-S and macroquartz cement in the sediments points to a formation during the advanced stages of sandstone diagenesis at elevated temperatures (70 to 150 °C), which is in contrast to the very low burial (in the range of a few hundred metres) proposed for the Valley of Lakes sediments. Thus, there is an apparent inconsistency between the actual literature and our results, particularly regarding the mineralogical and palaeo-climatic evolution of the Valley of Lakes sediments. We present here a new model for the post-depositional history of this famous study site, besides discussing implications for changes in the palaeo-climatic evolution reflected in the Valley of Lakes sediments.

### A revised model for the post-depositional evolution of the Valley of Lakes sediments

**Fig. 7:**
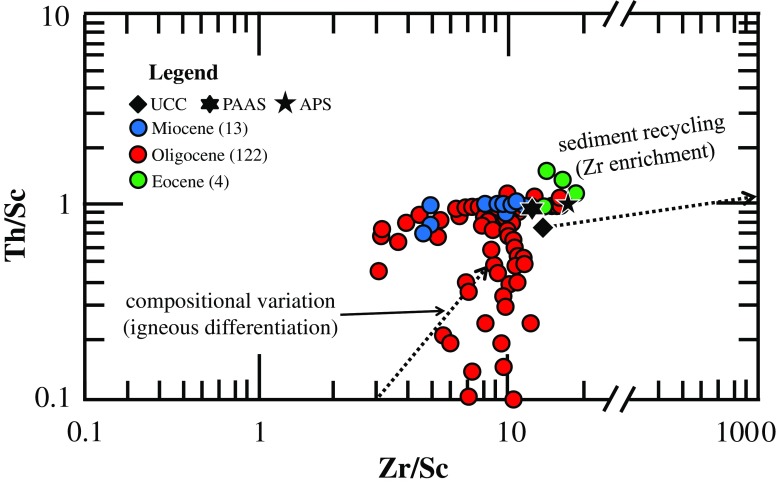
Th/Sc versus Zr/Sc cross-plot of McLennan (1993) demonstrating that the investigated samples from the Tsagaan Ovoo to Loh fms. plot well in the field of the upper crustal rocks and cluster around the compositions of the Upper Continental Crust (UCC), Average Proterozoic Shale (APS) and Post-Archean average Australian Shale (PAAS). This indicates that sediment recycling was insignificant (Bahlburg and Dobrzinski 2011)

The source rock composition, depositional environment and potential recycling processes can be identified using trace element composition data of bulk rock samples. Positive trends of the HREE with TiO_2_ and HFSE (Table 1) point to their incorporation in heavy minerals present in the Hsanda Gol and Loh fms. sediments. These heavy minerals have been delivered from metamorphic and magmatic source rocks exposed in the hinterland of the Valley of Lakes (McLennan et al. 1993). Based on gravel lithologies, Höck et al. (1999) demonstrated that the samples from the Tsagaan Ovoo to Loh fms. are predominated by quartz, pegmatite, granite, siltstone, basalt and carbonate clasts, with the heavy mineral fraction containing epidote, zircon, tourmaline, garnet, rutile, pyroxene, amphibole and sphene. Such a diamictic composition suggests a heterogeneous provenance of the gravel components being influenced by basic to intermediate, low- to medium-grade metamorphic and magmatic source rocks of Late Proterozoic to Late Permian age, which crop out in the catchment areas of the alluvial fans (Zorin et al. 1993). Despite the fact that the heavy mineral spectra are comparable throughout the entire Tsagaan Ovoo to Loh fms., recycling of older sediments and long transport distances are considered unlikely because multiple reworking-(re)deposition cycles typically result in a strong enrichment of Zr and in a depletion of Sc and Th. The Th/Sc vs. Zr/Sc cross-plot (Fig. 7) is a sensitive indicator for studying the source rock composition. This plot reveals that the investigated sediments have an upper crustal composition and were virtually not affected by post-depositional recycling processes. Even though an aeolian origin of the finest fraction cannot be ruled out based on the trace element data presented here, this finding suggests that the immature (arkosic) mud- to sandstones from the Hsanda Gol and Loh fms. were primarily derived from alluvial fans that have formed the upper Tsagaan Ovoo sediments. The latter feature is confirmed by remarkable lithological changes observed throughout the entire sedimentary succession (Fig. 3), which were interpreted to reflect changes in the depositional environment during the Eocene to Miocene. This is expressed in the Tsagaan Ovvo Fm. being dominated by braided fluvial fan and lake deposits (Höck et al. 1999: 92–100, Daxner-Höck et al. 2017, this issue), whereas the Hsanda Gol sediments represent ephemeral lake to braided fluvial fan deposits that are affected by re-sedimentation of the underlying Tsagaan Ovo Fm. (Badamgarav 1993; Höck et al. 1999). Finally, the Loh sediments are of similar composition but contain reworked lithoclasts from the basalt I and Hsanda Gol sediments (Daxner-Höck et al. 2017, this issue) and thus can be easily differentiated from the former.

However, a pre-condition required for the accurate interpretation of mineralogical as well as major and trace element signatures of bulk samples is the identification of distinct weathering paths (Zhang et al. 2014). Hydrolysis, leaching, sorting of sediments and diagenesis, for instance, result in distinct chemical, mineralogical and physical changes in the sediments and sedimentary rocks. Such processes have to be considered in the assessment of source rock compositions, depositional settings and sedimentary recycling processes and can be traced by means of the CIA, which provide a quantitative measure for tracing physical and chemical weathering (McLennan et al. 1993; Bauluz et al. 2000; Jin et al. 2006; Bahlburg and Dobrzinski 2011). The obtained CIA values, from 70 to 76, suggest that chemical weathering of the (arkosic) sandstones occurred during illitization (Fig. 4). This unidirectional weathering pathway can also be followed by the A-CN-K diagram (Fig. 6), where sorting, K-metasomatism and frictional variation in the provenance of sediments played only a minor role on affecting the bulk sediment’s mineralogical and geochemical signatures compared with their strong modification associated with the illitization (Nesbitt and Young 1984; Fedo et al. 1995; Yang et al. 2004; Bahlburg and Dobrzinski 2011).

The post-depositional alteration of the mostly (arkosic) mud- to sandstones need temperature around 70 to 150 °C. As the geological setting does not allow diagenesis through burying, we propose that hydrothermal fluids were the driving force for this diagenetic pathway. As samples post-basalt group II are affected in the same way than older ones, this hydrothermalism should have been active from Miocene to present. We thus propose that the latest illitization event co-occurred with the deposition of the basalt III during the middle Miocene at ~13 Ma, even if this basalt group has, today, a smaller geographic expansion than the Hsanda Gol Fm. The hydrothermal alteration of K-feldspar provided the K^+^ and Al^3+^ ions and the silicic acid to the interstitial solution, which are required both for the growth of discrete illite and I-S and for the syntaxial overgrowth of quartz as soon as a certain supersaturation threshold, with respect to these mineral phases, was reached in the interstitial solution (e.g. Hower et al. 1976; Bauer et al. 2000; Baldermann et al. 2012). This conceptual model is adequate to explain: (i) the predominance of illite and quartz (33 ± 8 and 35 ± 9 wt%, on average), (ii) the very low orthoclase content (2 ± 1 wt%, on average) observed throughout the sedimentary succession and (iii) the local restricted occurrences of zeolite and vermiculite associated with intensively weathered amphibole minerals originally present in basalt (Fig. 3). Kaolinite and chlorite could have also been formed by a dissolution–precipitation mechanism simultaneously and/or soon after the multi-stage illitization event(s), but they can be allochthonous in origin as well (Lanson et al. 1996; Beaufort et al. 2016). Even if we assume an allochthonous origin of kaolinite and chlorite, their occurrence in trace amounts (<2 wt%) contradicts the pronounced large aeolian deposition previously proposed by Sun and Windley (2015). In consequence, we cannot exclude local and short-distance aeolian contribution to the fluvial and lake deposit sedimentation, as it is common in arid to semi-arid environments (e.g. Xiao et al. 2010; Licht et al. 2014, 2016a), but apparently, we found no mineralogical and geochemical evidence for long distance transport and/or large aeolian deposition.

### Processes controlling the (isotope) geochemical composition of soil carbonates

The illitization resulted in a strong mineralogical and chemical overprinting of the Hsanda Gol to Loh sediments, implying that any palaeo-climatic information recorded in the paleosols could have been disturbed during post-depositional alteration. Moreover, the pristine δ^13^C and δ^18^O isotopic signatures of soil carbonate are often affected by exposure to meteoric or hydrothermal fluids (Marshall 1992), burial diagenesis (e.g. Marshall 1992; Kaufman and Knoll 1995) and oxidation of organic matter (Kent-Corson et al. 2009). Thus, palaeo-environmental reconstructions based on isotopic signatures of carbonate present in calcrete have to be interpreted carefully.

**Fig. 8:**
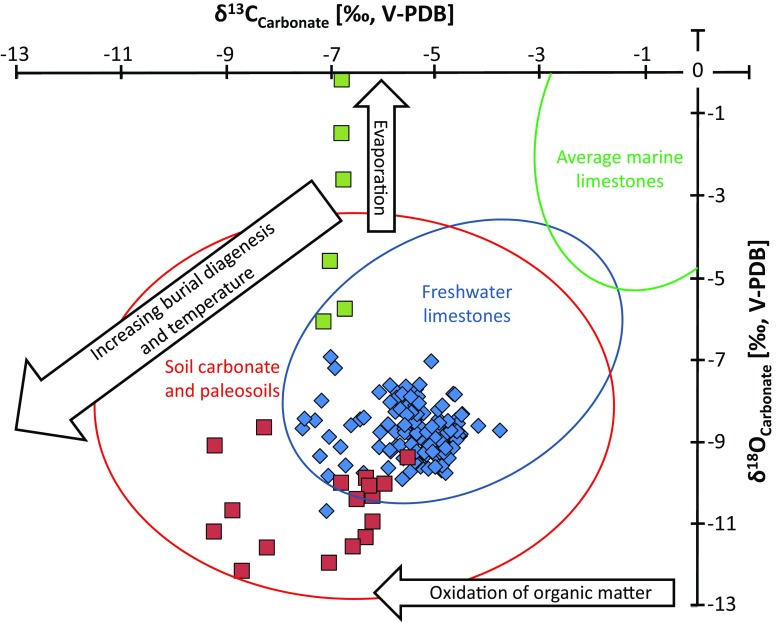
δ^13^C vs. δ^18^O cross plot. Soil carbonates collected in close vicinity to the basalt horizons (*red squares*) show some signs of alteration through interaction with hydrothermal fluids. Samples from the Lower Hsanda Gol Fm. (*green squares*) indicate formation in a playa-like environment. The majority of the samples (*blue rhombs*) preserved their pristine δ^13^C and δ^18^O isotope signature of paleosol formation and can be used for palaeo-climatic interpretations

**Fig. 9:**
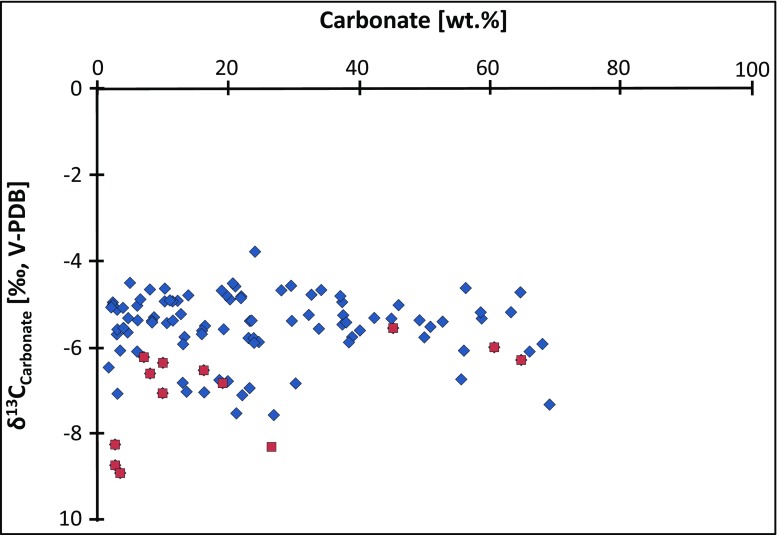
δ^13^C vs. total calcite (expressed as wt.% CaCO_3_) cross plot, showing that the vast majority of the soil carbonate is authigenic in origin and resistant to diagenetic overprinting. *Red squares* represent samples taken in close vicinity to the basalt groups I and II. See text for explanations

We propose, however, that the δ^13^C and δ^18^O isotope composition of soil carbonate was not disturbed by the post-depositional illitization event(s) and still records the climatic conditions prevailing during the precipitation of calcite from meteoric solutions, for the following reasons: (i) the δ^13^C and δ^18^O profiles should show a covariation in the case of strong diagenetic overprinting, which is apparently not the case here (Fig. 8), and (ii) δ^13^C and δ^18^O signatures are more prone to diagenetic alteration in a siliciclastic matrix than in pure carbonates (Kaufman and Knoll 1995). However, our isotopic values show no correlation with the total amount of carbonate present in the bulk samples (Fig. 9).

Soil carbonate frequently comprises of authigenic (i.e. calcrete), secondary (i.e. calcite spar) and detrital (i.e. lithoclasts) carbonate that may carry different δ^13^C and δ^18^O signatures. From a petrological point of view, the most widely absence of secondary calcite spar and dolomite, and the preservation of the crypto- to microcrystalline, honeycomb-like texture of calcite suggest that there is no other significant source of detrital and secondary calcite that could disturb the bulk δ^13^C and δ^18^O signatures of carbonate bound to paleosol horizons. In order to prove that the authigenic calcrete nodules and lenses account for ~100% of the soil carbonate, some samples were analysed for their δ^13^C and δ^18^O isotope bulk composition (Table 3). These values indicate that only few samples containing less than 10 wt% of calcite could be influenced by small abundances of detrital carbonate, but they do not blur the δ^13^C and δ^18^O signatures of the authigenic soil carbonate.

**Table 3:** δ^13^C and δ^18^O isotope composition measured on bulk rock and on calcrete nodules and lenses extracted from the same samples. These values indicate that only few samples with <10 wt% of calcite may be influenced by detrital and/or secondary carbonate minerals

	LECO	Calcrete		Bulk	
	CaCO_3_	δ^13^C	δ^18^O	δ^13^C	δ^18^O
	%	(‰), VPDB	(‰), VPDB	(‰), VPDB	(‰), VPDB
HTE/13a	6.9	−8.1	−10.2	−6.4	−9.7
HTE/12	51.2	−4.4	−8.6	−4.6	−8.5
HTE/11b	54.6	−5.4	−8.9	−5.3	−8.8
HTE/11a	3.8	−7.3	−11.0	−7.1	−10.7
HTE/9b	28.4	−5.1	−9.5	−5.2	−9.4
HTE/9a	3.8	−6.3	−11.2	−6.3	−11.3
HTE/8	5.0	−6.1	−10.6	−6.2	−10.9
HTE/7b	46.0	−5.7	−8.9	−5.5	−9.4
HTE/7a	2.8	−6.4	−11.7	−6.6	−11.5
HTE/6	11.8	−6.3	−10.3	−6.5	−10.4
HTE/1e	38.9	−5.9	−10.3	−6.2	−10.3
HTE/1d	4.9	−6.9	−11.7	−7.0	−11.9
HTE/1c	61.2	−6.2	−9.5	−6.0	−10.0
HTE/1a	67.2	−6.2	−10.1	−6.3	−10.1

The only exception is the carbonate samples collected in close vicinity to the prominent horizons of basalt groups I and II (Figs. 2 and 8, red squares), which display notably lighter δ^13^C (and δ^18^O for basalt II) values, compared with the rest of the calcrete samples (Figs. 2 and 8, blue rhombs). During volcanic activity associated with the development of the basalt groups, ^12^C–^12^C bonds break more easily than ^12^C–^13^C bonds, and the result is that residual carbonates, after vaporization, typically show more negative δ^13^C values than their precursor phase (Valley 1986; Richoz 2006). Thus, these samples (two samples taken below basalt I at the Taatsiin section and few samples collected at different levels around basalt II at the Hutuliin Teeg section) have not been considered in the climatic trends discussed below.

Considering, however, that most of the calcrete samples preserved their pristine δ^18^O and δ^13^C signatures of formation, palaeo-climatic trends can be interpreted based on changes in the δ^13^C curve (Fig. 2). The δ^13^C record of the soil carbonate is ultimately linked to variations in the isotopic composition of the soil solution from which the calcrete nodules and lenses have been precipitated (Schön et al. 2016). However, the δ^13^C isotopic composition of the soil solution can be modified in various ways. For instance, CO_2_ present in soil is a mixture of two isotopically distinct endmembers, namely atmospheric CO_2_ and CO_2_ derived from the in situ oxidation of organic matter present in soils (e.g. Khadikikar et al. 2000). Changes in the relative abundance of these isotopically different carbon pools and subsequent fractionation of the oxygen and carbon isotopes during (re)equilibration of CO_2_ with the soil solution greatly modify the δ^13^C signal of the precipitating calcite. Stratigraphic changes in δ^13^C_soil_ (and accordingly in the δ^13^C_carbonate_) values follow the: (i) variations in aridity, (ii) changes in the atmospheric *p*CO_2_ and (iii) changes in proportion between the C4/C3 photosynthesis pathways of plants. In our case, this last point cannot have any effect as C4 plants arrived in Mongolia not before late Miocene (Zhang et al. 2009; Edwards et al. 2010). From ~33 to 22 Ma, the atmospheric CO_2_ concentration decreased from 800 to 200 ppm (Zhang et al. 2013), which should be translated in a trend towards lighter δ^13^C_soil_ values. We do not see this trend in our data, and thus, changes in aridification in Central Mongolia may have overprinted this effect.

Increases in aridity can increase the δ^13^C of soil carbonate in three ways: (1) water stress induces a limitation of diffusion during photosynthesis, which increases the δ^13^C in plants (Kohn 2010); (2) a decrease in plant productivity, which increases the ratio of atmospheric CO_2_ to soil respired CO_2_ (Cerling 1984; Takeuchi et al. 2010); and (3) a shallowing of soil carbonate formation due to reduced infiltration, which also increases the ratio of atmospheric CO_2_ relative to the contribution of δ^13^C_soil_. The abovementioned processes result in an increase in δ^13^C (Caves et al. 2014), but the decrease in plant productivity is proven to have the largest effect (Cerling and Quade 1993; Caves et al. 2014). In summary, a positive excursion of the δ^13^C signature of soil carbonate (i.e. calcrete nodules) preserved in the paleosols of the Valley of Lakes sediments is ultimately linked to aridification.

δ^18^O values of pedogenic carbonates are mainly determined by the δ^18^O composition of the soil water, which in turn is strongly related to the δ^18^O composition of local meteoric water, being affected by soil temperature and evaporation rate (Cerling 1984; Quade et al. 1989; Li et al. 2016). It has been shown that in semi-arid and arid areas, the ratio soil water evaporation/amount of precipitation has the most significant effect (Quade and Cerling 1995; Li et al. 2016).

### Palaeo-climatic trends recorded in the Oligo-Miocene sediments

A palaeo-climatic reconstruction of the Oligocene to Miocene sediments from the Valley of Lakes is mainly established on the δ^13^C_carbonate_ profile (Fig. 2). A distinctive, positive δ^13^C isotope excursion (approximately −7.6 to −5.4‰ of δ^13^C, VPDB) occurs in the lower Rupelian (~31 Ma, lower to middle Hsanda Gol Fm., 40 to 62 m in Fig. 2), which can be assigned to a distinct early Oligocene aridification in Central Asia, a feature confirmed by the δ^18^O data. This aridification step has to be differentiated from the well-known Eocene–Olicocene Transition aridification event at ~33 Ma (Meng and McKenna 1998; Dupont-Nivet et al. 2007; Kraatz and Geisler 2010; Abels et al. 2011; Wang et al. 2012; Bosboom et al. 2014; Caves et al. 2014; Li et al. 2016) as it post-dated it clearly. This shift at ~31 Ma correspond to the boundary between Biozone A and B. The mammal assemblage, however, does not show significant changes in diversities or turnover rates (Harzhauser et al. 2016); the communities seem to be stable. Above a prominent sandstone bed at ~59 to 62 m, the δ^13^C signatures scatter in the range from approximately −5.9 to −4.6‰ of δ^13^C, VPDB, during the late Rupelian and the early Chattian without any significant variations in the carbon isotopic record. The transition between the biozones B and C is, however, marked by an important turnover in the mammalian community, mainly the rodents (Harzhauser et al. 2016), and corresponds to the OGM. This major climatic event is not well resolved in our record. Another significant shift towards heavier δ^13^C values (−6.2 to −4.8‰ of δ^13^C, VPDB, 88 to 93 m in Fig. 2) occurs at the top of the Hsanda Gol Fm., ~24 Ma, between the biozones C1 and C1-D. This trend is seen in the Taatssiin Gol section (TGR-C) and Hsanda Gol section (SHG), but is less clear in the Tatal Gol section (TAT), where the δ^13^C values scatter around −4.5 ± 0.4‰ already since the base of biozone C. This emphasises the very local response of the paleosols to climatic changes on different settings in the same area. This latter aridification corresponds to the prominent Late Oligocene Extinction (Harzhauser et al. 2016) and to the global Late Oligocene Warming (Paul et al. 2000). During the latest Oligocene to earliest Miocene, the δ^13^C (and δ^18^O) values show very strong variation (−10.5 to −4.5‰ of δ^13^C, VPDB, above 94 m in Fig. 2), which corresponds to an increased presence of cross-bedded fluvial deposits. This coarser lithology could have been more prone to diagenesis and late cement precipitation than the fine sediments of the Hsanda Gol Fm. Alternatively, a more humid climate during the formation of the Loh Fm. is certified by these fluvial sediments and changes in the gastropod record (Neubauer et al. 2013). These strong isotopic fluctuations could therefore reflect cyclic regional-scale climatic variations associated with the end of the global Late Oligocene Warming and the first Miocene Glacial period. The combination of our (isotope) geochemical, mineralogical, sedimentological and petrographic results leads us to suggest that long-term changes in the global and regional climate are partly well recorded in the investigated sedimentary succession of the Valley of Lakes. However, some global events like the OGM are not well documented in our records, and some aridification events (at ~31 and ~24 Ma) do not seem to correspond to any major global climatic change. Ongoing research and novel climate models are now required to better resolve global and regional scale palaeo-climate changes in Central Asia.

Variations in the δ^18^O values of soil carbonate throughout the lower Hsanda Gol Fm. (biozone A, 25 to 40 m in Fig. 2) are very large ( approximately −9.1 to −0.2‰ of δ^18^O, VPDB). Alternating extremely high to low evaporation degrees can produce such variability in the δ^18^O values (green squares in Fig. 8). This is in general agreement with the sedimentological data that point to sediment deposition in playas during the early Rupelian (Badamgarav 1993). Up-section, a trend towards heavier δ^18^O values (approximately −9.5 to −8.0‰ of δ^18^O, VPDB) was observed in the Rupelian (lower part of biozone B, 40 to 65 m in Fig. 2), which points to a distinct decrease in precipitation. This corresponds to the increase in aridification seen in our δ^13^C record. The δ^13^C peak is not seen at the transition between the Tibetan and the Chinese Loess Plateaus (Li et al. 2016). However, an increase in δ^18^O could be seen in the Lanzhou basin (Li et al. 2016) and in some section of the south Tarim Basin and of the Qaidam Basin (Kent-Corson et al. 2009). This discrepancy could be explained by changes in air-masses circulation, which have only localy an impact on soil productivity. This is followed by a shift towards progressively lighter δ^18^O data (approximately −8.0 to −9.0‰ of δ^18^O, VPDB) in the upper Rupelian (the upper part of biozone B). This could indicate an increase in precipitation which is not seen in the δ^13^C record or another change in air-masses circulation (Kent-Corson et al. 2009). Due to the large scatter in the δ^18^O values in the range from approximately −7 to −12‰ above ~75 m in the section (Fig. 2), potential palaeo-climatic changes recorded in the Upper Hsanda Gol to Loh fms. are not distinguishable based on δ^18^O data. This important scattering could be due to either climatic variability or increased diagenetic influences.

## Conclusions

Our novel mineralogical and (isotope) geochemical dataset of the highly fossiliferous Valley of Lakes sediments (Mongolia, Central Asia) suggests that intense illitization resulted in a strong overprinting of the mostly fluvial and lacustrine sediments of the Taatsiin Gol basin. The lath-like morphology and the 1 M polytype structure of the illite particles point to precipitation at elevated temperatures (70 to 150 °C), implying that the illitization was the result of circulating hydrothermal fluids generated during post-Oligocene basalt flood event(s). This hydrothermal circulation resulted in a significant post-depositional alteration and in an accompanying all encom-passing homogenization of the mineralogical and major element composition of the entire Eocene to Miocene succession. The trace element composition, i.e. Th/Sc and Zr/Sc ratios, however, reveals a short transportation distance of the sediments and little, if any, reworking-(re)deposition cycles. This confirms that the sediments of the Hsanda Gol to Loh fms. were mainly derived from the alluvial fans that have formed the later Tsaggan Ovoo sediments (Höck et al. 1999). Such short transportation distance and the huge abundance of authigenic illite and I-S contradict the large aeolian origin previously proposed for the fine fraction of the Hsanda Gol sediments (Sun and Windley 2015).

The crypto- to microcrystalline, honeycomb-like calcite present in calcrete nodules and lenses within abundant paleosol horizons shows, by contrast, an outstanding resistance against alteration and is therefore suitable to be used for palaeo-climatic reconstructions. In particular, the excursions towards less negative values in the δ^13^C record of the authigenic soil carbonate reveal a distinct early Oligocene aridification in the Valley of Lakes at ~31 Ma and a less intense (regional-scale) aridification at ~24 Ma that correlates with the Late Oligocene Extinction in mammalian communities. The Oligocene Glacial Maximum, which corresponds to an important faunal turnover in the Valley of Lakes, did not produce any significant imprint in our geochemical record. The Oligocene to Miocene Transition (OMT) is characterised by a higher variability in climate, which corresponds to the Late Oligocene Warming and the first Miocene Glacial. This record, which post-dates the retreat of the Tarim sea and predates the main tectonic up-lift events, confirm that aridification during Oligocene is a general feature for Central Asia (Licht et al. 2016b). These multiple aridification events generally follow global climatic trends; however, regional scale climatic and faunal variation is also recorded in the sediments of the Valley of Lakes.
